# A Super Fast Algorithm for Estimating Sample Entropy

**DOI:** 10.3390/e24040524

**Published:** 2022-04-08

**Authors:** Weifeng Liu, Ying Jiang, Yuesheng Xu

**Affiliations:** 1 Guangdong Province Key Laboratory of Computational Science, School of Computer Science and Engineering, Sun Yat-sen University, Guangzhou 510006, China; liuwf27@mail2.sysu.edu.cn; 2 Department of Mathematics and Statistics, Old Dominion University, Norfolk, VA 23529, USA; y1xu@odu.edu

**Keywords:** entropy, sample entropy, fast algorithm, Monte Carlo method

## Abstract

Sample entropy, an approximation of the Kolmogorov entropy, was proposed to characterize complexity of a time series, which is essentially defined as −log(B/A), where *B* denotes the number of matched template pairs with length *m* and *A* denotes the number of matched template pairs with m+1, for a predetermined positive integer *m*. It has been widely used to analyze physiological signals. As computing sample entropy is time consuming, the box-assisted, bucket-assisted, x-sort, assisted sliding box, and kd-tree-based algorithms were proposed to accelerate its computation. These algorithms require $O(N^{2})$ or $O(N^{2-\frac{1}{m+1}})$ computational complexity, where *N* is the length of the time series analyzed. When *N* is big, the computational costs of these algorithms are large. We propose a super fast algorithm to estimate sample entropy based on Monte Carlo, with computational costs independent of *N* (the length of the time series) and the estimation converging to the exact sample entropy as the number of repeating experiments becomes large. The convergence rate of the algorithm is also established. Numerical experiments are performed for electrocardiogram time series, electroencephalogram time series, cardiac inter-beat time series, mechanical vibration signals (MVS), meteorological data (MD), and 1/f noise. Numerical results show that the proposed algorithm can gain 100–1000 times speedup compared to the kd-tree and assisted sliding box algorithms while providing satisfactory approximate accuracy.

## 1. Introduction

Kolmogorov entropy is a well-suited measure for the complexity of dynamical systems containing noises. Approximate entropy (AppEn), proposed by Pincus [1], is an approximation of the Kolmogorov entropy. To overcome the biasedness of AppEn caused by self-matching, Richman proposed sample entropy (SampEn) [2] in 2000. SampEn is essentially defined as −log(B/A), where *B* denotes the number of matched template pairs with length *m* and *A* denotes the number of matched template pairs with m+1. SampEn has prevailed in many areas, such as cyber-physical systems, mechanical systems, health monitoring, disease diagnosis, and control. Based on AppEn and SampEn, multiscale entropy [3] and hierarchical entropy [4] were developed for measuring the complexity of physiological time series in multiple time scales. Since low-frequency filters are involved, multiscale entropy can weaken the influence of meaningless structures such as noise on complexity measurement. By adding the sample entropy of the high-frequency component of the time series, the hierarchical entropy provides more comprehensive and accurate information and improves the ability to distinguish different time series. Multiscale entropy, hierarchical entropy, and their variants have been applied to various fields such as fault identification [5,6] and feature extraction [7], beyond physiological time series analysis.

Computing SampEn requires counting the number of similar templates of time series. In other words, it requires counting the number of matched template pairs for a given time series. Clearly, direct computing of SampEn requires computational complexity of $O(N^{2})$, where *N* is the length of the time series analyzed. To accelerate the computation of SampEn, kd-tree based algorithms for sample entropy were proposed, which reduce the time complexity to $O(N^{2-\frac{1}{m+1}})$, where *m* is the template (also called pattern) length [8,9]. In addition, box-assisted [10,11], bucket-assisted [12], lightweight [13], and assisted sliding box (SBOX) [14] algorithms were developed. However, the complexity of all these algorithms is $O(N^{2})$. Recently, an algorithm proposed in [15] for computing approximate values of AppEn and SampEn, without theoretical error analysis, still requires $O(N^{2})$ computational costs in the worst scenario, even though it requires only O(N) number of operations in certain best cases. Developing fast algorithms for estimating SampEn is still of great interest.

The goal of this study is to develop a Monte-Carlo-based algorithm for calculating SampEn. The most costly step in computing SampEn is to compute the matched template ratio B/A of length *m* over length m+1. Noting that $\frac{A}{N(N-1)}$ (resp. $\frac{B}{N(N-1)}$) is the probability that templates of length *m* (resp. m+1) are matched, the ratio B/A can be regarded as a conditional probability. From this viewpoint, we can approximate this conditional probability of the original data set by that of a data set randomly down-sampled from the original one. Specifically, we randomly select $N_{0}$ templates of lengths *m* and $N_{0}$ templates of m+1 from the original time series. We then count the number $\tilde{A}$ (resp. $\tilde{B}$) of matched pairs among the selected templates of lengths *m* (resp. m+1). We repeat this process $N_{1}$ times, and compute the mean ${\bar{A}}_{N_{1}}$ (resp. ${\bar{B}}_{N_{1}}$) of $\tilde{A}$ (resp. $\tilde{B}$). Then, we use $-\log({\bar{B}}_{N_{1}}/{\bar{A}}_{N_{1}})$ to approximate −log(B/A) for the time series to measure its complexity. We establish the computational complexity and convergence rate of the proposed algorithm. We then study the performance of the proposed algorithm, by comparing it with the kd-tree-based algorithm and the SBOX method on the electrocardiogram (ECG) time series, electroencephalogram time series (EEG), cardiac inter-beat (RR) time series, mechanical vibration signals (MVS), meteorological data (MD), and 1/f noise. Numerical results show that the proposed algorithm can gain more than 100 times speedup compared to the SBOX algorithm (the most recent algorithm in the literature to the best of our knowledge) for a time series of length $2^{16}-2^{18}$, and more than 1000 times speedup for a time series of length $2^{19}-2^{20}$. Compared to the kd-tree algorithm, the proposed algorithm can again achieve up to 1000 times speedup for a time series of length $2^{20}$.

This article is organized in five sections. The proposed Monte-Carlo-based algorithm for estimating sample entropy is described in Section 2. Section 3 includes the main results of the analysis of approximate accuracy of the proposed algorithm, and the proofs are given in the Appendix A. Numerical results are presented in Section 4, and conclusion remarks are made in Section 5.

## 2. Sample Entropy via Monte Carlo Sampling

In this section, we describe a Monte-Carlo-based algorithm for estimating the sample entropy of a time series.

We first recall the definition of sample entropy. For all k∈N, let $Z_{k}:=\left\{1,2,\ldots ,k\right\}$. The distance of two real vectors $a:=[a_{l}:l\in Z_{k}]$ and $b:=[b_{l}:l\in Z_{k}]$ of length *k* is defined by

$$\rho \left(a,b\right):=\max\{|a_{l}-b_{l}|:l\in Z_{k}\}.$$

We let $u:=(u_{i}\in R:i\in Z_{n})$ be a time series of length n∈N. For m∈N, we let N:=n−m−1. We define a set *X* of *N* vectors by $X:=\left\{x_{i}:i\in Z_{N}\right\}$, where $x_{i}:=\left[u_{i+l-1}:l\in Z_{m}\right]$ is called a *template* of length *m* for the time series u. We also define a set *Y* of *N* vectors by $Y:=\left\{y_{i}:i\in Z_{N}\right\}$, where $y_{i}:=\left[u_{i+l-1}:l\in Z_{m+1}\right]$ is called a *template* of length m+1 for u. To avoid confusion, we call the elements in *X* and *Y* the templates for the time series u. We denote by #E the cardinality of a set *E*. We use $A_{i}$, $i\in Z_{N}$, to denote the cardinality of the set consisting of templates $x\in X\;\backslash \{x_{i}\}$ satisfying $\rho (x_{i},x)\leq r$, that is,

$$A_{i}:=\#\left\{x\in X\;\backslash \left\{x_{i}\right\}:\rho \left(x_{i},x\right)\leq r\right\}.$$

Likewise, for $i\in Z_{N}$, we let

$$B_{i}:=\#\left\{y\in Y\;\backslash \left\{y_{i}\right\}:\rho \left(y_{i},y\right)\leq r\right\}.$$

Letting

$$B:=\frac{1}{2}\sum_{i\in Z_{N}}B_{i}\;\;\mathrm{and}\;\;A:=\frac{1}{2}\sum_{i\in Z_{N}}A_{i},$$

we define the sample entropy of time series u by

$$\begin{array}{c} \mathrm{SampEn}\left(u,m,r\right):=\left\{\begin{array}{c} -\log\left(\frac{B}{A}\right),\;\mathrm{if}\;A>0,B>0,\\ -\log\left(\frac{2}{N(N-1)}\right),\;\mathrm{otherwise}. \end{array}\right. \end{array}$$

The definition of sample entropy yields the direct algorithm, which explicitly utilizes two nested loops, where the inner one computes $A_{i}$ and $B_{i}$, and the outer one computes *A* and *B*. Algorithm 1 will be called repeatedly in the Monte-Carlo-based algorithm to be described later.
**Algorithm 1** Direct method for range counting**Require:**  Sequence $u:=(u_{i}:i\in Z_{N+m})$, subset $s\subset Z_{N}$, template length *m* and threshold *r*.1: **procedure**  DirectRangeCounting (u,s,m,r)2:     Set count=0,3:     Set L=#s,4:     **for** i=1 to *L* **do**5:         Set $a=[u_{s_{i}+l-1}:l\in Z_{m}]$,6:         **for** j=i+1 to *L* **do**7:            Set $b=[u_{s_{j}+l-1}:l\in Z_{m}]$,8:            **if** ρ(a−b)≤r **then**9:                count=count+1,10:     **return** count

The definition of sample entropy shows that sample entropy measures the predictability of data. Precisely, in the definition of sample entropy, B/A measures a conditional probability that when the distance of two templates a and b is less than or equal to *r*, the distance of their corresponding (m+1)-th component is also less than or equal to *r*. From this perspective, we can approximate this conditional probability of the original data set by computing it on a data set randomly down-sampled from the original one. To describe this method precisely, we define the notations as follows.

We choose a positive integer $N_{0}$, randomly draw $N_{0}$ numbers from $Z_{N}$ without replacement, and form an $N_{0}$-dimensional vector. All of such vectors form a subset Ω of the product space

$$Z_{N}^{N_{0}}:=Z_{N}⊗Z_{N}⊗\cdots ⊗Z_{N}\;\;\left(N_{0}\text{-folds}\right),$$

that is,

$$\Omega :=\{s:=\left[s_{1},\cdots ,s_{N_{0}}\right]\in Z_{N}^{N_{0}}:s_{i}\neq s_{j}\;\mathrm{for}\;\mathrm{all}\;i\neq j\}.$$

Suppose that F is the power set of Ω (the set of all subsets of Ω, including the empty set and Ω itself). We let *P* be the uniform probability measure satisfying P(s)=1/(#Ω) for all s∈Ω and define the probability space {Ω,F,P}. The definition of Ω implies $\#\Omega =\frac{N!}{(N-N_{0})!}$, and thus the probability measure satisfies $P\left(s\right)=\frac{(N-N_{0})!}{N!}$ for all s∈Ω. The definition of F means all events that may occur in the sample space Ω are considered in the probability space {Ω,F,P}. We randomly select $N_{0}$ templates of length *m* and $N_{0}$ templates of length m+1 from the original time series. We then count the number $\tilde{A}$ (resp. $\tilde{B}$) of matched pairs among the selected templates of lengths *m* (resp. m+1). That is,

$$\tilde{A}\left(s\right):=\frac{1}{2}\#\left\{\left(i,j\right):i,j\in Z_{N_{0}}\;\mathrm{with}\;i\neq j,\;\mathrm{and}\;\rho \left(x_{s_{i}},x_{s_{j}}\right)\leq r\right\},\;s\in \Omega ,$$

and

$$\tilde{B}\left(s\right):=\frac{1}{2}\#\left\{\left(i,j\right):i,j\in Z_{N_{0}}\;\mathrm{with}\;i\neq j,\;\mathrm{and}\;\rho \left(y_{s_{i}},y_{s_{j}}\right)\leq r\right\},\;s\in \Omega .$$

We repeat this process $N_{1}$ times.

Note that $\tilde{A}$ and $\tilde{B}$ are random variables on the probability space {Ω,F,P}. Let ${\bar{A}}_{N_{1}}$ and ${\bar{B}}_{N_{1}}$ be the averages of random variables $\tilde{A}$ and $\tilde{B}$, respectively, over the $N_{1}$ repeated processes. That is,

$${\bar{A}}_{N_{1}}:=\frac{1}{N_{1}}\sum_{k=1}^{N_{1}}\tilde{A}\left(s_{k}\right),\;\mathrm{and}\;\;{\bar{B}}_{N_{1}}:=\frac{1}{N_{1}}\sum_{k=1}^{N_{1}}\tilde{B}\left(s_{k}\right),$$

where $\left\{s_{k}:k\in Z_{N_{1}}\right\}$ is a subset of Ω. With ${\bar{A}}_{N_{1}}$ and ${\bar{B}}_{N_{1}}$, we can estimate the sample entropy −log(B/A) by computing $-\log({\bar{B}}_{N_{1}}/{\bar{A}}_{N_{1}})$. We summarize the procedure for computing $-\log({\bar{B}}_{N_{1}}/{\bar{A}}_{N_{1}})$ in Algorithm 2 and call it *the Monte-Carlo-based algorithm for evaluating sample entropy* (MCSampEn). In MCSampEn, $s_{k}$, $k\in Z_{N_{0}}$, are selected by the Hidden Shuffle algorithm proposed in [16].
**Algorithm 2** Monte-Carlo-based algorithm for evaluating sample entropy**Require:** Sequence $u=(u_{i}:i\in Z_{N+m})$, template length *m*, tolerance r∈R, sample size $N_{0}$ and number of experiments $N_{1}$, probability space {Ω,F,P}1: **procedure** MCSampEn ($u,m,r,N_{0},N_{1}$)2:     Set ${\bar{A}}_{N_{1}}=0$ and ${\bar{B}}_{N_{1}}=0$,3:     **for** k=1 to $N_{1}$ **do**4:         Select $s_{k}\in \Omega$, randomly, with uniform distribution,5:         Compute $\tilde{A}\left(s_{k}\right)$ by calling DirectRangeCounting ($u,s^{\left(k\right)},m,r$),6:         Compute $\tilde{B}\left(s_{k}\right)$ by calling DirectRangeCounting ($u,s^{\left(k\right)},m+1,r$),7:         ${\bar{A}}_{N_{1}}={\bar{A}}_{N_{1}}+\frac{1}{N_{1}}\tilde{A}\left(s^{\left(k\right)}\right)$,8:         ${\bar{B}}_{N_{1}}={\bar{B}}_{N_{1}}+\frac{1}{N_{1}}\tilde{B}\left(s^{\left(k\right)}\right)$,9:      $entropy=-\log\frac{{\bar{B}}_{N_{1}}}{{\bar{A}}_{N_{1}}}$,10:     **return** entropy

We next estimate the computational complexity of MCSampEn measured by the number of arithmetic operations. To this end, we recall Theorem 3.5 of [16] which gives the number of arithmetic operations used in the Hidden Shuffle algorithm.

**Theorem** **1.** *The Hidden Shuffle algorithm generates a random sample of size $N_{0}$ sequentially from a population of size N with $O(N_{0})$ arithmetic operations in total.*

**Theorem** **2.** *The total number of arithmetic operations needed in Algorithm 2 is $O(N_{1}\left(N_{0}^{2}+N_{0}\right))$.*

**Proof.**  For each $k\in Z_{N_{1}}$, according to Theorem 1, the number of arithmetic operations needed for selecting $s^{\left(k\right)}$ on line 4 of Algorithm 2 is $O(N_{0})$. Moreover, from Algorithm 1 we can see that for each $k\in Z_{N_{1}}$, the number of arithmetic operations needed for computing $\tilde{A}\left(s_{k}\right)$ and $\tilde{B}\left(s_{k}\right)$ on lines 5 and 6 is $O(N_{0}^{2})$. Thus, by counting the number of arithmetic operations needed for lines 7, 8, and 9 of Algorithm 2, we obtain the desired result. □

Theorem 2 indicates that the computational complexity of MCSampEn is controlled by setting appropriate sampling parameters $N_{0}$ and $N_{1}$. When $N_{0}$ and $N_{1}$ are fixed, the computational complexity of MCSampEn is independent of the length *N* of time series u. Meanwhile, we can also select $N_{0}$ and $N_{1}$ depending on *N* to balance the error and computational complexity of MCSampEn. For example, we can set $N_{0}:=\max\left\{1024,\left\lfloor \sqrt{N}\right\rfloor \right\}$ and $N_{1}:=\min\left\{5+\log_{2}N,\left\lfloor N/N_{0}\right\rfloor \right\}$, where ⌊a⌋ denotes the greatest integer no bigger than a∈R. In this case, the computational complexity is $O(N\log_{2}N)$.

Noting that MCSampEn provides an approximation of the sample entropy, and not the exact value, convergence of MCSampEn is an important issue. We will discuss this in Section 3.

## 3. Error Analysis

In this section, we analyze the error of MCSampEn. Specifically, we will establish an approximation rate of MCSampEn in the sense of almost sure convergence.

A sequence of $\left\{V_{k}:k\in N\right\}$ of random variables in probability space {Ω,F,P} is said to converge almost surely to V∈{Ω,F,P}, denoted by

$$V_{k}\overset{a.s.}{\to }V,$$

if there exists a set N∈F with P(N)=0 such that for all ω∈Ω\N,

$$\lim_{k\to \infty }V_{k}\left(\omega \right)=V\left(\omega \right).$$

It is known (see [17]) that $\left\{V_{k}:k\in N\right\}$ converges almost surely to V∈{Ω,F,P} if and only if

$$\lim_{k\to +\infty }P\left(\left\{\underset{i\geq k}{\sup}\left|V_{i}-V\right|>\epsilon \right\}\right)=0,\;\;\mathrm{for}\;\mathrm{all}\;\;\epsilon >0.$$

Furthermore, we can describe the convergence rate of $\left\{V_{i}:i\in N\right\}$ by the declining rate of the sequence $\left\{P\left(\left\{\sup_{i\geq k}\left|V_{i}-V\right|>\epsilon \right\}\right):k\in N\right\}$ for all ϵ>0. If for α>0,

$$P\left(\left\{\underset{i\geq k}{\sup}\left|V_{i}-V\right|>\epsilon \right\}\right)=O\left(k^{-\alpha }\right),\;\;\mathrm{for}\;\mathrm{all}\;\;\epsilon >0,$$

we say $\left\{V_{i}:i\in N\right\}$ converges to *V* almost surely with rate α.

To establish the approximation error of MCSampEn, we first derive two theoretical results for the expectations and variations of $\frac{\tilde{A}}{N_{0}\left(N_{0}-1\right)}$ and $\frac{\tilde{B}}{N_{0}\left(N_{0}-1\right)}$. Then, by combining these results with the results of the almost sure convergence rate in [18] and the local smoothness of logarithm functions, we obtain the approximation rate of $\left\{-\log\left({\bar{B}}_{N_{1}}/{\bar{A}}_{N_{1}}\right):N_{1}\in N\right\}$ in the sense of almost sure convergence, which is the main theoretical result of this paper. We state these results below and postpone their proofs to the Appendix A.

The expectations of $\frac{\tilde{A}}{N_{0}\left(N_{0}-1\right)}$ and $\frac{\tilde{B}}{N_{0}\left(N_{0}-1\right)}$ are given in the following theorem.

**Theorem** **3.** *It holds that for all $N_{0}\in Z_{N}$ with $N_{0}>1$,*

(1) $$E\left[\frac{\tilde{A}}{N_{0}\left(N_{0}-1\right)}\right]=\frac{A}{N(N-1)},$$

*and*

(2) $$E\left[\frac{\tilde{B}}{N_{0}\left(N_{0}-1\right)}\right]=\frac{B}{N(N-1)}.$$

The next theorem presents the variations of $\frac{\tilde{A}}{N_{0}\left(N_{0}-1\right)}$ and $\frac{\tilde{B}}{N_{0}\left(N_{0}-1\right)}$.

**Theorem** **4.** *It holds that for all $N_{0}\in Z_{N}$ with $N_{0}>1$,*

(3) $$\mathrm{Var}\left[\frac{\tilde{A}}{N_{0}\left(N_{0}-1\right)}\right]=\frac{C_{N_{0}}}{N_{0}},$$

*and*

(4) $$\mathrm{Var}\left[\frac{\tilde{B}}{N_{0}\left(N_{0}-1\right)}\right]=\frac{C_{N_{0}}}{N_{0}},$$

*where*

(5) $$\begin{array}{ccc} C_{N_{0}} & := & \frac{B}{\left(N_{0}-1\right)N\left(N-1\right)}+\frac{N_{0}-2}{\left(N_{0}-1\right)N\left(N-1\right)\left(N-2\right)}\left(\sum_{l=1}^{N}B_{l}^{2}-2B\right)\\ & & +\frac{\left(N_{0}-2\right)\left(N_{0}-3\right)}{\left(N_{0}-1\right)N\left(N-1\right)\left(N-2\right)\left(N-3\right)}\left(B^{2}-\sum_{l=1}^{N}B_{l}^{2}+B\right)-\frac{N_{0}B^{2}}{N^{2}{\left(N-1\right)}^{2}}. \end{array}$$

*Moreover, there is $0<C_{N_{0}}<1+\frac{1}{2(N_{0}-1)}$.*

Based on Theorems 3 and 4, we can obtain $\log\frac{{\bar{B}}_{N_{1}}}{{\bar{A}}_{N_{1}}}\overset{a.s.}{\to }\log\frac{B}{A}$ by the Kolmogorov strong law of large numbers and the continuous mapping theorem. However, in practice it is desirable to quantify the approximation rate in the sense of almost sure convergence, so that we can estimate the error between $\log\frac{{\bar{B}}_{N_{1}}}{{\bar{A}}_{N_{1}}}$ and $\log\frac{B}{A}$. To this end, we define $\tau_{A}:=E\left[\left|\frac{\tilde{A}}{N_{0}\left(N_{0}-1\right)}-\frac{A}{N(N-1)}\right|\right]$, and $\tau_{B}:=E\left[\left|\frac{\tilde{B}}{N_{0}\left(N_{0}-1\right)}-\frac{B}{N(N-1)}\right|\right]$. Let $\gamma_{A}:=\frac{A}{2N(N-1)e}$ and $\gamma_{B}:=\frac{B}{2N(N-1)e}$. For all β>1 and 0<ϵ<1, we also let

(6) $$n_{\epsilon ,\beta }:=\max\left\{6\epsilon^{-1},\exp\left({\left(9\beta^{-1}\epsilon^{-1}\right)}^{\beta^{-1}/\left(1-\beta^{-1}\right)}\right)\right\}.$$

With the notation defined above, we present below the main theoretical result of this paper, which gives the rate of $\left\{-\log\frac{{\bar{B}}_{k}}{{\bar{A}}_{k}}:k\in N\right\}$ approximating $-\log\frac{B}{A}$ in the sense of almost sure convergence.

**Theorem** **5.** *Let β>1 and $N_{0}\in Z_{N}$ with $N_{0}>3$. If A,B>0, then there exist constants $D_{\beta }$ and ${\tilde{D}}_{\beta }$ (depending only on β) such that for all 0<ϵ<1 and $N_{1}>n_{\epsilon ,\beta }$, such that*

(7) $$\begin{array}{ccc} & & P\left(\underset{k>N_{1}}{\sup}\left|\log\frac{{\bar{B}}_{k}}{{\bar{A}}_{k}}-\log\frac{B}{A}\right|>\max\left\{\tau_{A},\tau_{B}\right\}\epsilon \right)\\ & \leq & \frac{72C_{N_{0}}}{\epsilon^{2}N_{0}N_{1}}\left(D_{\beta }+{\tilde{D}}_{\beta }{\left(\log N_{1}\right)}^{\beta -1}\right)\left(\frac{1}{\tau_{A}^{2}\gamma_{A}^{2}}+\frac{1}{\tau_{B}^{2}\gamma_{B}^{2}}\right). \end{array}$$

The proof for Theorems 3–5 are included in the Appendix A. Note that Theorem 5 indicates that $-\log\frac{{\bar{B}}_{k}}{{\bar{A}}_{k}}$ approximates $-\log\frac{B}{A}$ in the sense of almost sure convergence of order 1.

## 4. Experiments

We present numerical experiments to show the accuracy and computational complexity of the proposed algorithm MCSampEn.

As sample entropy has been prevalently used in a large number of areas, we consider several series with a variety of statistical features, including the electrocardiogram (ECG) series, RR interval series, electroencephalogram (EEG) series, mechanical vibration signals (MVS), meteorological data (MD), and 1/f noise. The ECG and EEG data can be downloaded from PhysioNet, a website offering access to recorded physiologic signals (PhysioBank) and related open-source toolkits (PhysioToolkit) [19]. The MVS data can be found in [20] and the website of the Case Western Reserve University Bearing Data Center [21]. The MD data can be downloaded from the website of the Royal Netherlands Meteorological Institute [22]. The databases used in this paper include:

**Long-Term AF Database (ltafdb)** [23]. This database includes 84 long-term ECG recordings of subjects with paroxysmal or sustained atrial fibrillation (AF). Each record contains two simultaneously recorded ECG signals digitized at 128 Hz with 12-bit resolution over a 20 mV range; record durations vary but are typically 24 to 25 h.
**Long-Term ST Database (ltstdb)** [24]. This database contains 86 lengthy ECG recordings of 80 human subjects, chosen to exhibit a variety of events of ST segment changes, including ischemic ST episodes, axis-related non-ischemic ST episodes, episodes of slow ST level drift, and episodes containing mixtures of these phenomena.
**MIT-BIH Long-Term ECG Database (ltecg)** [19]. This database contains 7 long-term ECG recordings (14 to 22 h each), with manually reviewed beat annotations.
**BIDMC Congestive Heart Failure Database (chfdb)** [25]. This database includes long-term ECG recordings from 15 subjects (11 men, aged 22 to 71, and 4 women, aged 54 to 63) with severe congestive heart failure (NYHA class 3–4).
**MGH/MF Waveform Database (mghdb)** [26]. The Massachusetts General Hospital/ Marquette Foundation (MGH/MF) Waveform Database is a comprehensive collection of electronic recordings of hemodynamic and electrocardiographic waveforms of stable and unstable patients in critical care units, operating rooms, and cardiac catheterization laboratories. Note that only the ECG records were considered in our experiments.
**RR Interval Time Series (RR).** The RR interval time series are derived from healthy subjects (RR/Health), and subjects with heart failure (RR/CHF) and atrial fibrillation (RR/AF).
**CHB-MIT Scalp EEG Database (chbmit)** [27]. This database contains (EEG) records of pediatric subjects with intractable seizures. The records are collected from 22 subjects, monitored for up to several days.
**Gearbox Database (gearbox)** [20]. The gearbox dataset was introduced in [20] and was published on https://github.com/cathysiyu/Mechanical-datasets (accessed on 27 March 2022).
**Rolling Bearing Database (RB)** [21]. This database as a standard reference for the rolling bearing fault diagnosis is provided by the Case Western Reserve University Bearing Data Center [21].
**Meteorological Database (MD)** [22]. The meteorological database used in this section records the hourly weather data in the past 70 years in the Netherlands.

**Figure 1:**
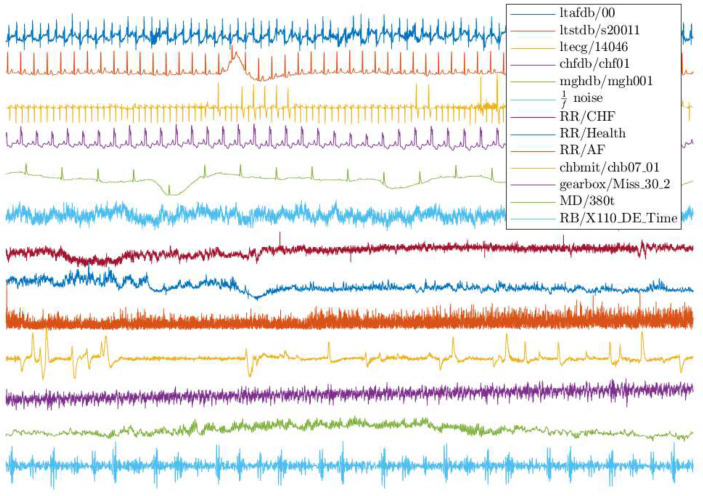
Samples of the dataset records.

As each database consists of multiple records from different subjects, we select one record randomly from each database. Specifically, we choose record “00” from ltafdb, “s20011” from ltstdb, “14046” from ltdb, “chf01” from chfdb, “mgh001” from mghdb, “chb07_01” from chbmit, “Miss_30_2” from gearbox, “XE110_DE_Time” from RB, and “380_t” from MD. Moreover, 1/f noise signal, an artificial signal, is studied to increase diversity. The time series considered in this section are illustrated in Figure 1, where all samples are normalized to have a standard deviation of 1, since the parameter threshold *r* is proportional to the standard deviation of the records, and thus the whole range of the records is negligible.

### 4.1. Approximation Accuracy

In the experiments presented in this subsection, we examine the approximation accuracy of the MCSampEn algorithm. Specifically, we set r:=0.15 and m:=4,5. We vary the sampling size $N_{0}$ and the number $N_{1}$ of computations to study the approximation accuracy of the proposed algorithm. In this experiment, records with lengths exceeding $10^{6}$ are truncated to have length $10^{6}$; otherwise, the entire records are used. Since in the MCSampEn algorithm, $s_{k}\in \Omega$ are selected randomly, the outcome of the algorithm depends on the selected value of $s_{k}$. To overcome the effect of the randomness, for every specified pair of $\left(N_{0},N_{1}\right)$, we run the algorithm 50 times and calculate the mean errors **(MeanErr)** and the root mean squared errors **(RMeanSqErr)** of the 50 outcomes.

**Figure 2:**
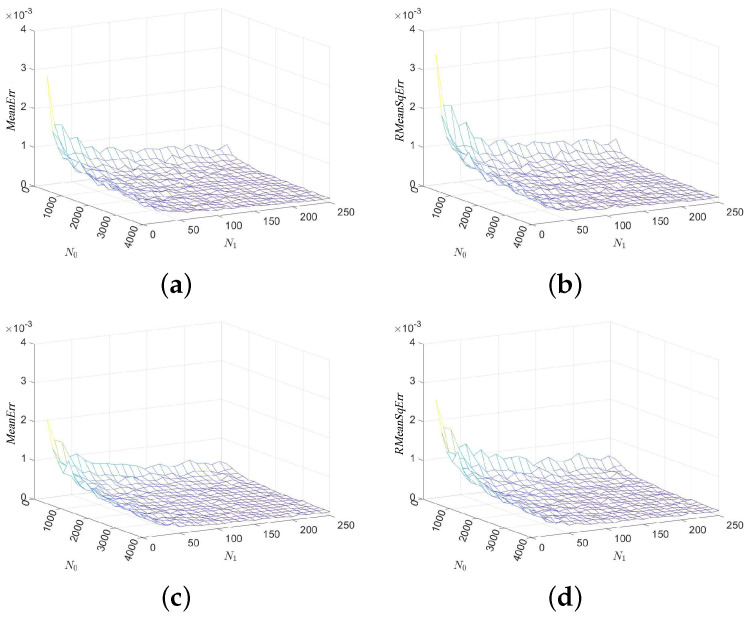
The values of MeanErr and RMeanSqErr for time series “mghdb/mgh001” with respect to the sample size $N_{0}$ and the number of computations $N_{1}$, where parameters r=0.15 and m=4,5. (**a**) MeanErr with m=4. (**b**) RMeanSqErr with m=4. (**c**) MeanErr with m=5. (**d**) RMeanSqErr with m=5.

In our first experiment, we consider series “mghdb/mgh001”, select parameters $N_{0}\in \left\{200i:i\in Z_{20}^{+}\right\}$, $N_{1}\in \left\{10i:i\in Z_{25}^{+}\right\}$, and show in Figure 2 the mean errors and the root mean squared errors of the MCSampEn outputs as surfaces in the $N_{0}$-$N_{1}$ coordinate system. Images (a) and (c) of Figure 2 show the values of MeanErr and images (b), (d), and (f) of Figure 2 show the values of RMeanSqErr. Figure 2 clearly demonstrates that both the mean errors and the root mean squared errors of the MCSampEn outputs converge to 0 as $N_{0}$ or $N_{1}$ increases to infinity. This is consistent with our theoretical analysis in the previous section.

**Figure 3:**
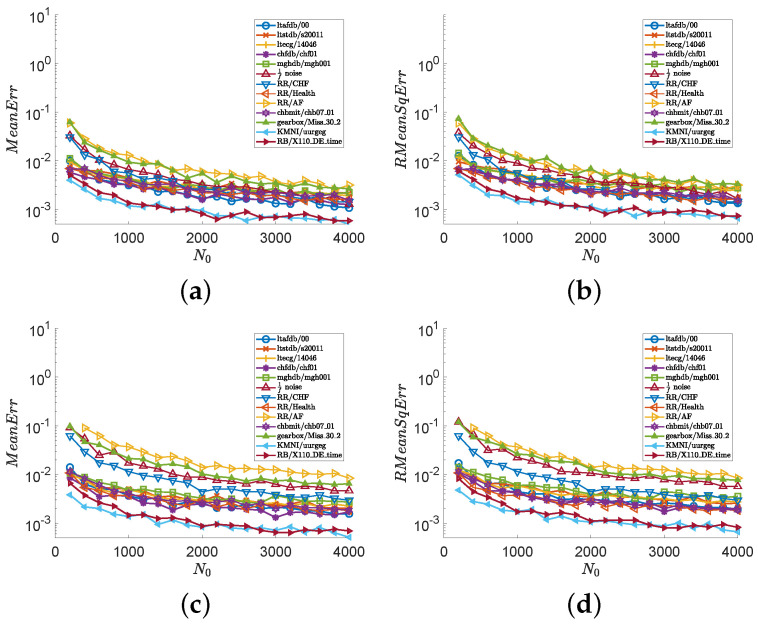
The values of MeanErr and RMeanSqErr with respect to $N_{0}\in \left\{200i:i\in Z_{20}^{+}\right\}$ and $N_{1}=150$, where parameters r=0.15 and m=4,5. (**a**) MeanErr with m=4. (**b**) RMeanSqErr with m=4. (**c**) MeanErr with m=5. (**d**) RMeanSqErr with m=5.

**Figure 4:**
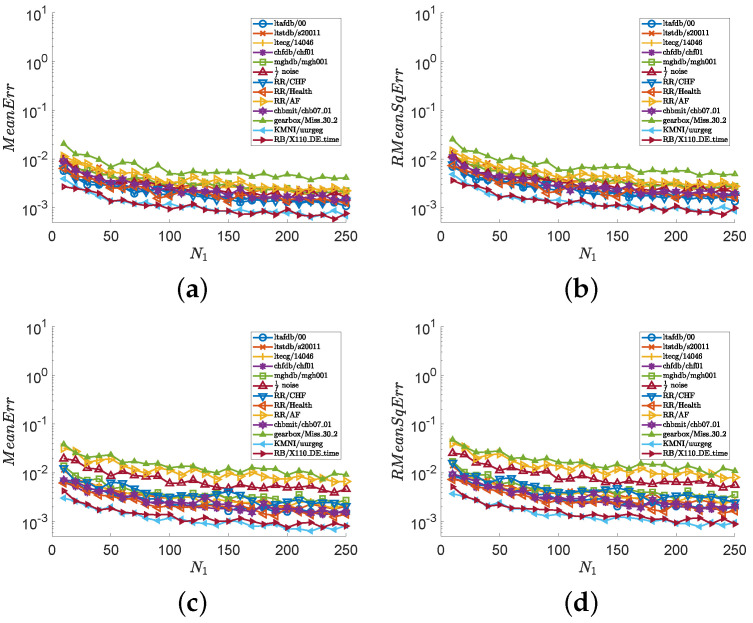
The values of MeanErr and RMeanSqErr with respect to $N_{0}=2\times 10^{3}$ and $N_{1}\in \left\{10i:i\in Z_{25}^{+}\right\}$, where parameters r=0.15 and m=4,5. (**a**) MeanErr with m=4. (**b**) RMeanSqErr with m=4. (**c**) MeanErr with m=5. (**d**) RMeanSqErr with m=5.

In the second experiment, we consider all series illustrated in Figure 1 and show numerical results in Figure 3 and Figure 4. Images (a), (c), and (e) of Figure 3 show the values of MeanErr, and images (b), (d), and (f) of Figure 3 show the values of RMeanSqErr, with $N_{0}\in \left\{200i:i\in Z_{20}^{+}\right\}$ and fixed $N_{1}=250$. Images (a), (c), and (e) of Figure 4 show the values of MeanErr, and images (b), (d), and (f) of Figure 4 show the values of RMeanSqErr, with $N_{0}=4000$ and $N_{1}\in \left\{10i:i\in Z_{25}^{+}\right\}$. Figure 3 indicates that the outputs of the MCSampEn algorithm converge as $N_{0}$ increases. We can also see from Figure 3 that when $N_{0}\geq 1500$, $N_{1}=150$, and m=4, both MeanErr and RMeanSqErr are less than $1\times 10^{-2}$ for all tested time series. In other words, the MCSampEn algorithm can effectively estimate sample entropy when $N_{0}\geq 1500$, $N_{1}=150$, and m=4. From Figure 4, we can also observe that the outputs of the MCSampEn algorithm converge as $N_{1}$ increases. This is consistent with the theoretical results established in Section 3.

We next explain how the randomness of a time series effects the accuracy of the MCSampEn algorithm by applying the algorithm to the stochastic process MIX(p), which has been widely applied to studies of sample entropy [1,2,28]. The MIX(p) is defined as follows. Let $x_{j}:=\alpha^{-1/2}\sin\left(12\pi j/12\right)$ for all $j\in Z_{N}$ where

$$\alpha :=\left(\sum_{j=1}^{12}\sin^{2}\left(2\pi j/12\right)\right)/12.$$

Let $\left\{y_{j}:j\in Z_{N}\right\}$ be a family of independent identically distributed (i.i.d) real random variables with uniform probability density on the interval $\left[-\sqrt{3},\sqrt{3}\right]$. Note that $\left\{x_{j}:j\in Z_{N}\right\}$ and $\left\{y_{j}:j\in Z_{N}\right\}$ are sequences with contrary properties: the former is a completely regular sine sequence, and the latter is completely random. Let p∈[0,1], and $\left\{z_{j}:j\in Z_{N}\right\}$ be a family of i.i.d random variables satisfying $z_{j}=1$ with probability *p* and $z_{j}=0$ with probability 1−p. Then, the MIX(p) process is defined as $\left\{m_{j}:=\left(1-z_{j}\right)x_{j}+z_{j}y_{j}:j\in Z_{N}\right\}$. It’s not hard to find that the parameter *p* controls the ratio of sine sequence and random noise in the MIX(p) process and the increase in *p* makes the MIX(p) process more random. When p=0, the MIX(p) process is a deterministic sine sequence. Meanwhile, when p=1, the MIX(p) process turns out completely unpredictable uniform noise. This feature makes it an ideal series to study how randomness affects the accuracy of the MCSampEn algorithm.

**Figure 5:**
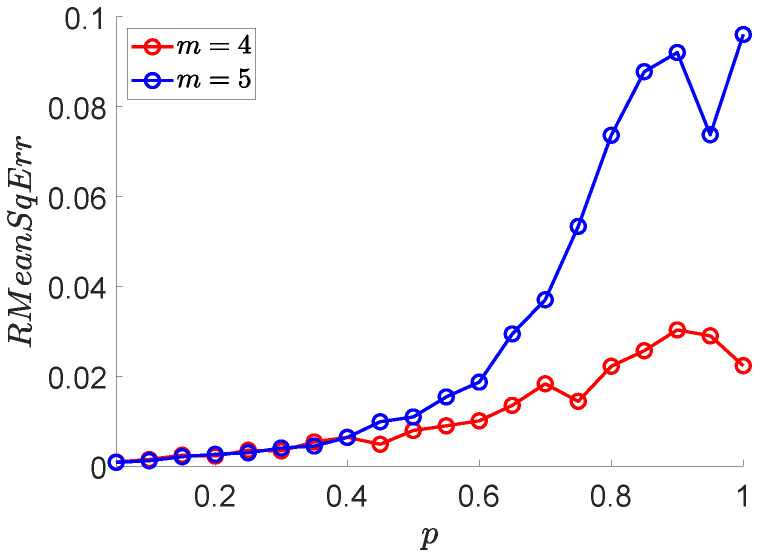
The values of RMeanSqErr with respect to *p*, where parameters r=0.15, m=4,5, $N=2^{20}$, $N_{0}=2000$, and $N_{1}=150$.

Here, we apply MCSampEn to MIX(p), $p\in \{0.5+0.5i:i\in Z_{19}\}$ and show the results of RMeanSqErr versus *p* in Figure 5. From Figure 5, we can observe that the values of RMeanSqErr increase linearly with a very small growth rate when p≤0.5. When p>0.5, the values of RMeanSqErr are significantly faster than that of p≤0.5. Therefore, we believe that when the randomness of a time series is weak, the error of the MCSampEn algorithm is small; as the randomness of the time series increases, the error of the MCSampEn grows.

### 4.2. Time Complexity

In the experiments presented in this subsection, we compare the computing time of the MCSampEn algorithm with that of the kd-tree algorithm [8] and SBOX algorithm [14], under the condition that the value of sample entropy computed by the MCSampEn algorithm is very close to the ground truth value. The computational time experiments are performed on a desktop computer running Windows 11, with an Intel(R) Core(TM) i5-9500 CPU, and 32GB RAM. The implementations of the kd-tree-based algorithm and the MCSampEn algorithm are available on the website https://github.com/phreer/fast_sampen_impl.git (accessed on 30 March 2022). As for the SBOX method, we utilize the implementation given by the original author, published on website https://sites.google.com/view/yhw-personal-homepage (accessed on 25 October 2021). To demonstrate the validity of the MCSampEn algorithm, we also show both the sample entropy estimated by MCSampEn and the corresponding ground truth.

As we have discussed above, the time complexity of the MCSampEn algorithm depends on the parameters $N_{0}$ and $N_{1}$. In this subsection, we discuss two strategies for choosing $N_{0}$ and $N_{1}$:

**S1**  Choose $N_{0}$ and $N_{1}$ to be independent of *N*, for example $N_{0}=2\times 10^{3}$ and $N_{1}=150$.
**S2**  Choose $N_{0}=\max\left\{1024,\left\lfloor \sqrt{N}\right\rfloor \right\}$ and $N_{1}=\min\left\{5+\log_{2}N,\left\lfloor N/N_{0}\right\rfloor \right\}$, depending on *N*.

An intuitive explanation of the second strategy is shown below. We would like to choose $N_{0}$ and $N_{1}$ such that the overall time complexity of executing the algorithm is O(NlogN). For this purpose, we expect $N_{0}$ to grow like $\sqrt{N}$ and $N_{1}$ to grow logarithmically in *N*. However, when *N* is not large enough, lack of sampling templates can seriously impair the accuracy of the algorithm. To overcome this problem, we set a lower bound of $N_{0}$ to 1024, which is a good trade-off between accuracy and time complexity. The experimental results in this subsection show that this strategy can produce satisfactory output even when *N* is small.

**Figure 6:**
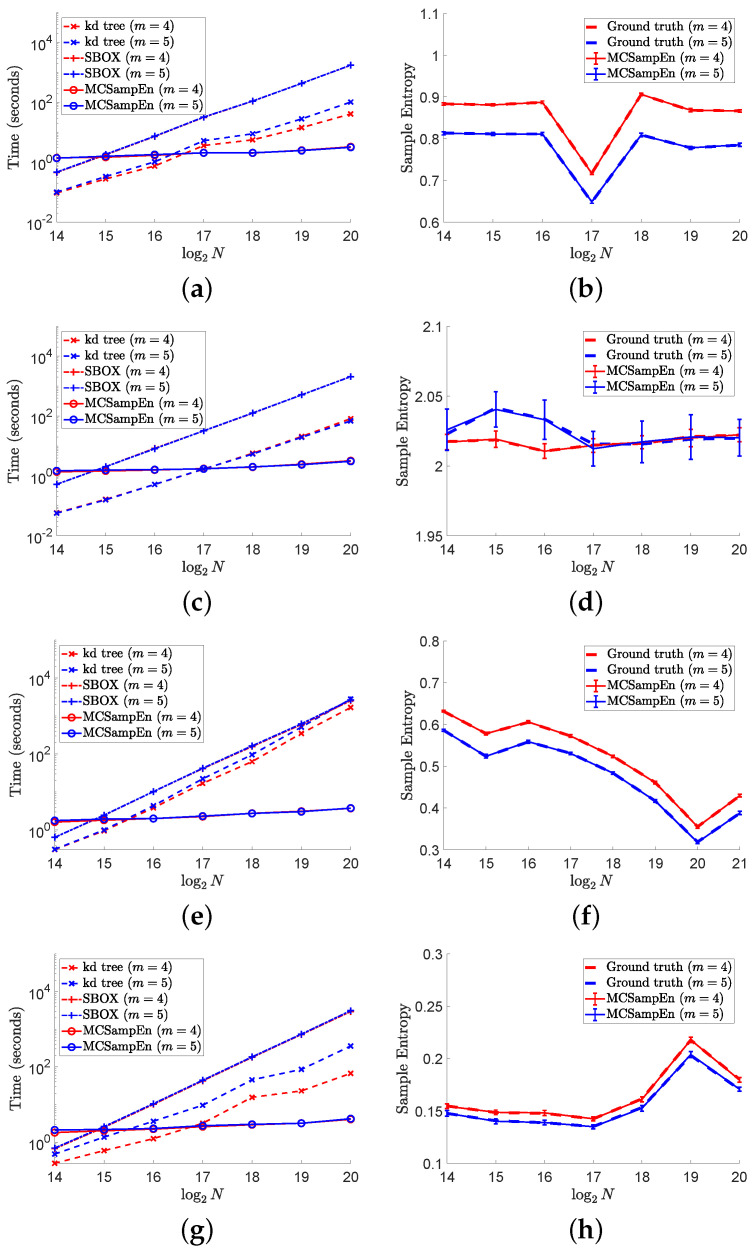
The left column shows the results of computational time versus data length *N* on different signals. In the right column, the values of RMeanSqErr are presented by error bars “I”, where the larger the value of RMeanSqErr, the longer the error bar “I”. In this figure, we set m=4,5, $N_{0}=2\times 10^{3}$, and $N_{1}=150$. (**a**) Time for “ltafdb/00”. (**b**) Sample entropy “ltafdb/00”. (**c**) Time for 1/f noise. (**d**) Sample entropy for 1/f noise. (**e**) Time for “chbmit/chb07_01”. (**f**) Sample entropy for “chbmit/chb07_01”. (**g**) Time “ltecg/14046”. (**h**) Sample entropy for “ltecg/14046”.

The results on different signals “ltafdb/00”, “1/f noise”, “chbmit/chb07_01”, and “ltecg/14046” are shown in Figure 6, where the first strategy is adopted by setting $N_{0}=2\times 10^{3}$ and $N_{1}=150$, and the results for m=4 are marked by red color, and the results for m=5 are marked by blue. In the left column of Figure 6, the values of computation time consumed by the kd-tree, SBOX, and MCSampEn algorithms are plotted, respectively, with the dashed lines marked “x”, the dash-dot lines marked “+”, and the solid lines marked “o”. From the results shown in the left column of Figure 6, we can find that MCSampEn is faster than the SBOX algorithm when *N* is greater than $2^{15}$. We also can see when the time series “chbmit/chb07_01” and “ltecg/14046” have length *N* of $2^{20}$, MCSampEn is nearly 1000 times faster than the SBOX algorithm. Compared to the kd-tree algorithm, the MCSampEn algorithm can still achieve up to hundreds of times acceleration when $N=2^{20}$. In addition, the time complexity of MCSampEn algorithm is close to a constant relative to *m*, and is much smaller than the kd-tree and SBOX algorithms when *N* is large enough. Meanwhile, the computational time (shown in the left column of Figure 6) required is hardly affected by the times series length *N*.

The right column of Figure 6 shows the average of 50 outputs of the MCSampEn algorithm for different time series under the settings of $N_{0}=2\times 10^{3}$ and $N_{1}=150$, where the red solid lines plot the average for the cases of m=4, and the blue solid lines plot the average for the cases of m=5. In the right column of Figure 6, the values of ground truth for the cases of m=4 and m=5 are plotted by the red and blue dashed lines, respectively. Meanwhile, in the right column of Figure 6, we use error bars “I” to represent the values of RMeanSqErr, where the larger the value of RMeanSqErr, the longer the error bar “I”. From the length of error bar “I”, we can see that the values of RMeanSqErr are small compared to the ground truth. Especially on the time series “ltafdb/00”, “chbmit/chb_0701”, and “ltecg/14046”, the values of RMeanSqErr are negligible compared to the values of ground truth. These results imply that when $N_{0}=2\times 10^{3}$ and $N_{1}=150$, the sample entropy estimated by the MCSampEn algorithm can effectively approximate the ground truth value.

**Figure 7:**
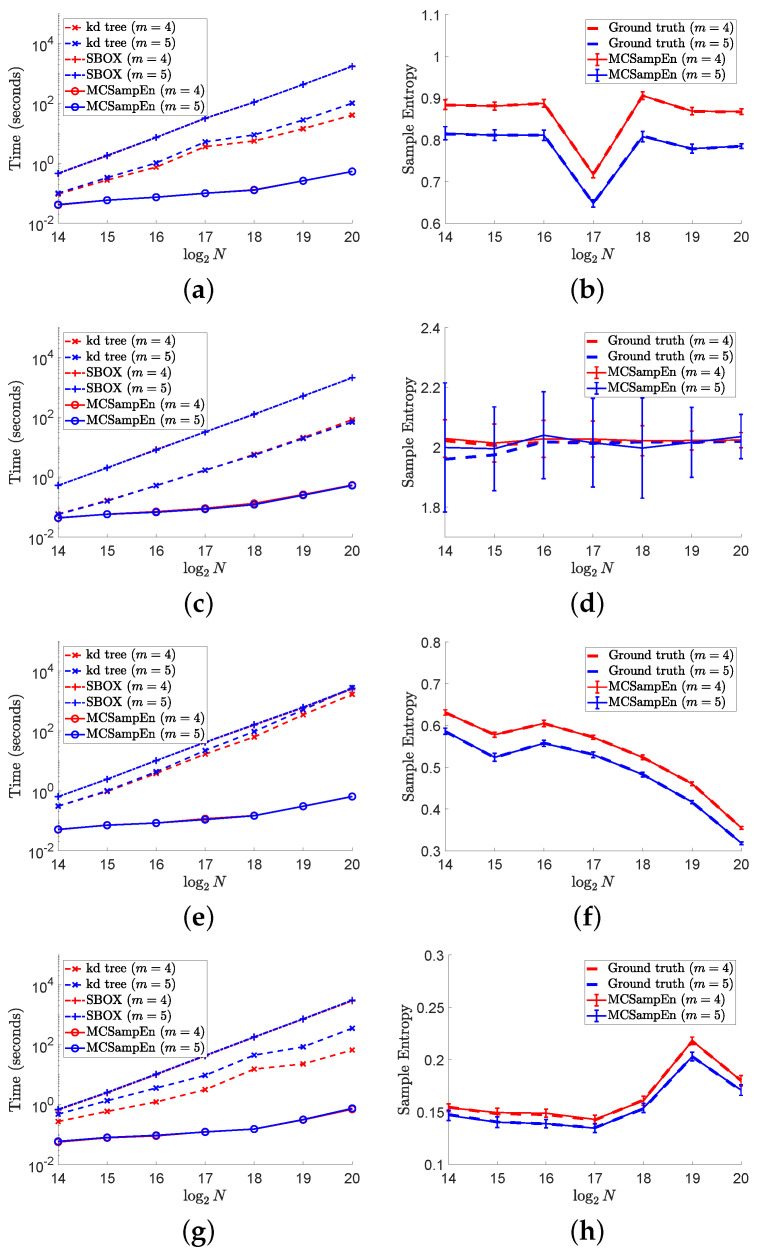
The left column shows the results of computational time versus data length *N* on different signals. The right column shows the values of RMeanSqErr by error bar, where the larger the value of RMeanSqErr, the longer the error bar “I”. In this figure, we set m=4,5, $N_{0}=\max\left\{1024,\left\lfloor \sqrt{N}\right\rfloor \right\}$, and $N_{1}=\max\left\{1,\left\lfloor N/N_{0}\right\rfloor \right\}$. (**a**) Time for “ltafdb/00”. (**b**) Sample entropy “ltafdb/00”. (**c**) Time for 1/f noise. (**d**) Sample entropy for 1/f noise. (**e**) Time for “chbmit/chb07_01”. (**f**) Sample entropy for “chbmit/chb07_01”. (**g**) Time “ltecg/14046”. (**h**) Sample entropy for “ltecg/14046”.

The results of the second strategy are shown in Figure 7, where $N_{0}=\max\left\{1024,\left\lfloor \sqrt{N}\right\rfloor \right\}$ and $N_{1}=\min\left\{5+\log_{2}N,\left\lfloor N/N_{0}\right\rfloor \right\}$. The results for m=4 are marked by red color, and the results for m=5 are marked by blue color. The left column of Figure 6 shows the values of computation time consumed by the kd-tree, SBOX, and MCSampEn algorithms, which are presented by the dashed lines marked “x”, the dash-dot lines marked “+”, and the solid lines marked “o”, respectively. From the left column of Figure 7, we also can see that with the second strategy, the computational time of MCSampEn algorithm is much less than that of the kd-tree and SBOX algorithms, since the computational complexity of Algorithm 2 is O(NlogN). Furthermore, we observe that MCSampEn achieves a speedup of more than 100 compared to the SBOX algorithm when *N* goes from $2^{16}$ to $2^{18}$, and it is over 1000 times faster when $N=2^{20}$. Compared to the kd-tree algorithm, the MCSampEn algorithm can still obtain up to 1000 times acceleration when $N=2^{20}$.

In the right column of Figure 7, we plot the average of 50 outputs of the MCSampEn algorithm for different time series by the red and blue solid lines for m=4 and m=5, respectively. At the same time, the values of ground truth for the cases of m=4 and m=5 are plotted by the red and blue dashed lines, respectively. As in Figure 6, we use the error bar “I” to represent the values of RMeanSqErr. Comparing the error bar “I” in Figure 6, we can see that the values of the RMeanSqErr in this experiment are larger than that shown in Figure 6. However, the value of RMeanSqErr is still small in terms of the values of ground truth. Moreover, we can observe that the length of the error bars decreases as *N* increases. This means that we can obtain a better approximation of sample entropy as the time series length increases.

**Figure 8:**
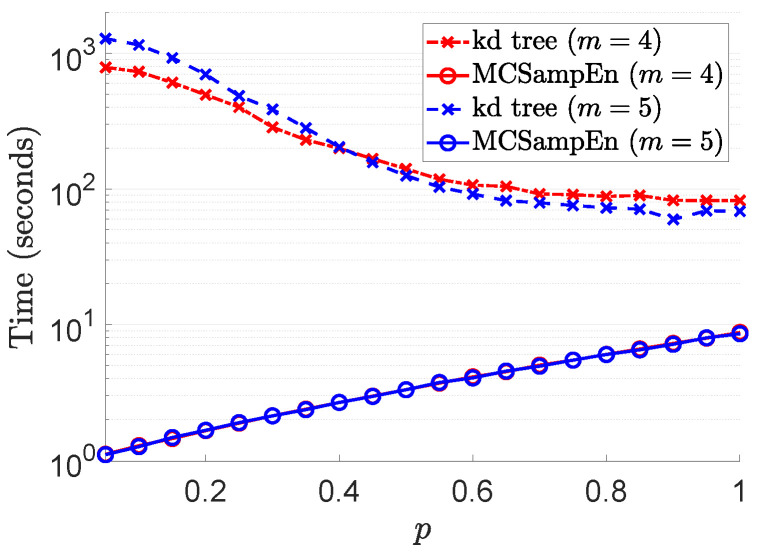
The results of computational time with respect to *p*, where parameters r=0.15, m=4,5, $N=2^{20}$, $N_{0}$, and $N_{1}$ are selected such that relative error RMeanSqErr/SampEn≤0.02.

To reveal the effect of randomness on the speedup, we compare the time taken by the kd-tree and MCSampEn algorithms to compute the sample entropy of the time series MIX(p), $p\in \{0.5+0.5i:i\in Z_{19}\}$. The experimental results are shown in Figure 8, where the results for m=4 are marked by red color, and the results for m=5 are marked by blue. The values of computation time consumed by the kd-tree and MCSampEn algorithms are plotted, respectively, with the dashed lines marked “x” and the solid lines marked “o”. In this experiment, we set $N=2^{20}$ and r=0.15. We also let $N_{0}=1000+3000p$ and $N_{1}=80+70p$ to ensure that the relative error RMeanSqErr/SampEn is no greater than 0.02. From Figure 8, we can see that when the value of *p* is less than 0.2, compared with the kd-tree algorithm, the MCSampEn algorithm can achieve 300 to 1000 times speedup. When the value of *p* is greater than 0.8, our algorithm can still obtain a 10x speedup relative to the kd-tree algorithm.

From the experiments in this subsection, we can observe that the MCSampEn algorithm can achieve a high speedup when it is applied to different types of signals. In fact, compared with kd-tree algorithm, the MCSampEn algorithm can achieve high accuracy and more than 300 times acceleration when the time series has less randomness. When the randomness of the time series is high, our algorithm can still obtain a speedup of nearly 10 times.

### 4.3. Memory Usage

**Figure 9:**
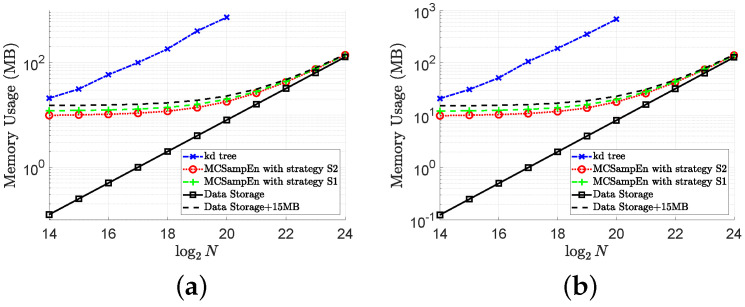
The results of memory usage versus data length *N* with m=4,5. (**a**) Memory usage for m=4. (**b**) Memory usage for m=5.

In order to show the performance of the MCSampEn algorithm more comprehensively, we also compare the memory usage of the kd-tree and MCSampEn algorithms. The memory usage on signal “ltstdb/s20011” is shown in Figure 9, where the memory usage for m=4 and m=5 is shown in Figure 9a,b, respectively. In this figure, the memory usage of the kd-tree algorithm is plotted by the blue dash-dot lines marked “x”. The memory usage of the MCSampEn algorithm with the first and second strategies is plotted by the green dashed lines marked “+” and the red dotted lines marked “o”, respectively. In Figure 9, the first strategy is adopted by setting $N_{0}=2048$ and $N_{1}=150$, and the second strategy is adopted by $N_{0}=\max\left\{1024,\left\lfloor \sqrt{N}\right\rfloor \right\}$ and $N_{1}=\min\left\{5+\log_{2}N,\left\lfloor N/N_{0}\right\rfloor \right\}$. We also present the memory usage for storing the data by the black solid lines marked “□”.

From the results shown in Figure 9, it can be seen that when the size of the data is $2^{20}$, the memory required by the kd-tree algorithm is almost 36 times that of the memory required by the MCSampEn algorithm. This is because the kd-tree algorithm requires a large memory space to save the kd-tree. Meanwhile, the experimental results in Figure 9 also show that the amount of memory required by the MCSampEn algorithm is only about 15 MB more than the amount of memory required to store the data when the length of data is between $2^{14}$ and $2^{24}$. This is because the MCSampEn algorithm requires additional memory for storing $N_{0}$ templates and to execute the subroutines that generate random numbers.

Because the MCSampEn algorithm is based on Monte Carlo sampling and the law of large numbers, it is an easily parallelizable algorithm. Therefore, combined with distributed storage techniques, the idea of the MCSampEn algorithm can be used to compute sample entropy for large-scale data (for example, where the size of data is larger than 1 TB). Parallel algorithms for computing sample entropy of large-scale data will be our future work.

## 5. Conclusions

In this paper, we propose a Monte-Carlo-based algorithm called MCSampEn to estimate sample entropy and prove that the outputs of MCSampEn can approximate sample entropy in the sense of almost sure convergence of order 1. We provide two strategies to select the sampling parameters $N_{0}$ and $N_{1}$, which appear in MCSampEn. The experiment results show that we can flexibly select the parameters $N_{0}$ and $N_{1}$ to balance the computational complexity and error. From the experimental results, we can observe that the computational time consumed by the proposed algorithm is significantly shorter than the kd-tree and SBOX algorithms, with negligible loss of accuracy. Meanwhile, the computational complexity of our MCSampEn method is hardly affected by the time series length *N*. We also study how the randomness of the time series affects the accuracy and computation time of the MCSampEn algorithm by applying the algorithm to the stochastic process MIX(p). The results indicate that the proposed algorithm performs well for time series with less randomness.

## Data Availability

The data used are included in the article.

## Appendix A

In this Appendix, we provide proofs of Theorems 3–5, where Theorems 3 and 4 describe the expectations and variances of $\frac{\tilde{A}}{N_{0}\left(N_{0}-1\right)}$ and $\frac{\tilde{B}}{N_{0}\left(N_{0}-1\right)}$, and Theorem 5 presents the convergence rate of $\left\{-\log\frac{{\bar{B}}_{k}}{{\bar{A}}_{k}}:k\in N\right\}$.

Note that the only difference in the definitions between $\frac{\tilde{A}}{N_{0}\left(N_{0}-1\right)}$ and $\frac{\tilde{B}}{N_{0}\left(N_{0}-1\right)}$ is the template length. Without loss of generality, we discuss the expectation (2) and variation (4) of $\frac{\tilde{B}}{N_{0}\left(N_{0}-1\right)}$. The Equations (1) and (3) of $\frac{\tilde{A}}{N_{0}\left(N_{0}-1\right)}$ can be obtained in a similar way.

To analyze the expectation of $\frac{\tilde{B}}{N_{0}\left(N_{0}-1\right)}$, we define the following notation. For all $j\in Z_{N_{0}}$, we define random variable ${\tilde{B}}_{j}$ on the probability space {Ω,F,P} by

(A1) $${\tilde{B}}_{j}\left(s\right):=\#\left\{i\in Z_{N_{0}}:i\neq j\;\mathrm{and}\;\rho \left(y_{s_{i}},y_{s_{j}}\right)\leq r\right\},\;s\in \Omega .$$

For all $j\in Z_{N_{0}}$, the definition of ${\tilde{B}}_{j}$ indicates that ${\tilde{B}}_{j}\left(s\right)$ is the number of elements in $\left\{y_{s_{i}}:i\in Z_{N_{0}}\right\}$ that satisfy $\rho (y_{s_{i}},y_{s_{j}})\leq r$ and i≠j. From the definitions of $\tilde{B}$ and ${\tilde{B}}_{j}$, we have that for all s∈Ω,

$$\tilde{B}\left(s\right)=\frac{1}{2}\sum_{j=1}^{N_{0}}{\tilde{B}}_{j}\left(s\right).$$

For p,q,l∈N, we say random variable *V* follows the hypergeometric distribution H(p,q,l) if and only if the probability of V=k

Pr(V=k)=qkp−ql−qpl,ifmax{0,q+l−p}≤k≤min{q,l},0,otherwise.

See Section 5.3 of [29] for more details about the hypergeometric distribution. For all $l\in Z_{N}$, let $B_{l}:=\left\{i\in Z_{N}:i\neq l\;\mathrm{and}\;\rho \left(y_{i},y_{l}\right)\leq r\right\}$, which is the index set of elements of *Y* satisfying $\rho (y_{i},y_{l})\leq r$. From the definition of $B_{l}$, we have that $B_{l}=\#B_{l}$. For the purpose of analyzing the expectation of $\frac{\tilde{B}}{N_{0}\left(N_{0}-1\right)}$, we recall the expectation of the hypergeometric distribution H(p,q,l) (see Theorem 5.3.2 in [29]) and prove a technical lemma as follows.

**Theorem** **A1.**
*For p,q,l∈N, the expectation of the hypergeometric distribution H(p,q,l) is $\frac{ql}{p}.$*

**Lemma** **A1.**
*Let $N_{0}\in Z_{N}$ with $N_{0}>1$. For any fixed $j\in Z_{N_{0}}$ and $l\in Z_{N}$, the conditional probability distribution of ${\tilde{B}}_{j}$ given $s_{j}=l$ is the hypergeometric distribution $H(N-1,B_{l},N_{0}-1)$. Moreover, for all $j\in Z_{N_{0}}$, the expectation of random variable ${\tilde{B}}_{j}$ is*

(A2)
$$E\left[{\tilde{B}}_{j}\right]=\frac{2(N_{0}-1)}{N(N-1)}B.$$

**Proof.** Let $j\in Z_{N_{0}}$ and $l\in Z_{N}$. From the definition of ${\tilde{B}}_{j}$, we can see that for all s∈Ω with $s_{j}=l$, ${\tilde{B}}_{j}\left(s\right)\leq \min\left\{B_{l},N_{0}-1\right\}$. On the other hand, since for all s∈Ω with $s_{j}=l$,

$$\left\{i\in Z_{N_{0}}:\rho \left(y_{s_{i}},y_{l}\right)>r\right\}\subset \left\{i\in Z_{N}:\rho \left(y_{i},y_{l}\right)>r\right\},$$

from the definitions of ${\tilde{B}}_{j}$ and $B_{l}$, we have that $N_{0}-{\tilde{B}}_{j}\leq N-B_{l}$. Thus, we can see that for all s∈Ω with $s_{j}=l$, $\max\left\{0,N_{0}-N+B_{l}\right\}\leq {\tilde{B}}_{j}\leq \min\left\{N_{0}-1,B_{l}\right\}$. This means that for $k<\max\{0,N_{0}-N+B_{l}\}$ or $k>\min\{N_{0}-1,B_{l}\}$,

$$\#\{s\in \Omega :{\tilde{B}}_{j}\left(s\right)=k\;\mathrm{and}\;s_{j}=l\}=0.$$

Meanwhile, it can be checked that for all s∈Ω with $s_{j}=l$ and $\max\left\{0,N_{0}-N+B_{l}\right\}\leq k\leq \min\left\{N_{0}-1,B_{l}\right\}$, ${\tilde{B}}_{j}\left(s\right)=k$ if and only if vector s contains *k* components belonging to $B_{l}$, and $N_{0}-1-k$ components belonging to $Z_{N}/\left(B_{l}\cup \left\{l\right\}\right)$. Note that there are Blk ways of drawing *k* elements from set $B_{l}$, and N−1−BlN0−1−k ways of drawing $N_{0}-1-k$ elements from set $Z_{N}/\left(B_{l}\cup \left\{l\right\}\right)$. Thus, by noting that each element in Ω is a permutation formed by extracting $N_{0}$ numbers from $Z_{N}$, we have that for all $\max\left\{0,N_{0}-N+B_{l}\right\}\leq k\leq \min\left\{N_{0}-1,B_{l}\right\}$,

(A3) #{s∈Ω:B˜j(s)=kandsj=l}=(N0−1)!BlkN−1−BlN0−1−k.

Note that $\#\left\{s\in \Omega :s_{j}=l\right\}=\frac{(N-1)!}{(N-N_{0})!}$, and the elements in $\left\{s\in \Omega :s_{j}=l\right\}$ are of equal probability. Hence, dividing the right term of (A3) by $\frac{(N-1)!}{(N-N_{0})!}$, we obtain

PB˜j=k∣sj=l=BlkN−1−BlN0−1−BlN−1N0−1,max{0,N0−N+Bl}≤k≤min{N0−1,Bl},0,otherwise.

This indicates that the conditional probability distribution of ${\tilde{B}}_{j}$ given $s_{j}=l$ is the hypergeometric distribution $H(N-1,B_{l},N_{0}-1)$ (see [29]). Since the conditional probability distribution of ${\tilde{B}}_{j}$ given $s_{j}=l$ is the hypergeometric distribution $H(N-1,B_{l},N_{0}-1)$, from Theorem A1 we have for any $j\in Z_{N_{0}}$ and $l\in Z_{N}$, $E\left[{\tilde{B}}_{j}|s_{j}=l\right]=\frac{B_{l}\left(N_{0}-1\right)}{N-1}$. Thus, by noting $\sum_{l\in Z_{N}}B_{l}=2B$ and $P\left(s_{j}=l\right)=\frac{1}{N}$ for all $l\in Z_{N}$, from the law of total expectation we obtain (A2). □

**The proof for Theorem 3 is shown as follows.**

**Proof.** From the definitions of $\tilde{B}$ and ${\tilde{B}}_{j}$, we know

(A4) $$E\left[\tilde{B}\right]=\frac{1}{2}\sum_{j=1}^{N_{0}}E\left[{\tilde{B}}_{j}\right].$$

Substituting (A2) into (A4) leads to (2). □

Next we consider the variance of $\frac{\tilde{B}}{N_{0}\left(N_{0}-1\right)}$. Since $\tilde{B}=\frac{1}{2}\sum_{j\in Z_{N_{0}}}{\tilde{B}}_{j}$, the variance of $\frac{\tilde{B}}{N_{0}\left(N_{0}-1\right)}$ can be obtained by summing the covariances $E[{\tilde{B}}_{j_{1}}{\tilde{B}}_{j_{2}}]$, $j_{1},j_{2}\in Z_{N_{0}}$. This motivates us to compute these covariances. As a preparation, we establish two auxiliary lemmas. For all $k,l\in Z_{N}$ with k≠l, we define $B_{kl}:=B_{k}\cap B_{l}$ and $B_{kl}:=\#B_{kl}$.

**Lemma** **A2.**
*It holds that*

(A5)
$$\sum_{k\in Z_{N}}\sum_{l\in Z_{N}\backslash \left\{k\right\}}B_{kl}=\sum_{l\in Z_{N}}B_{l}^{2}-2B.$$

**Proof.** Note that $B_{kl}⋂B_{k^{\prime }l^{\prime }}$ is not necessarily empty for $\left(k,l\right)\neq \left(k^{\prime },l^{\prime }\right)$. For $B_{kl}$, we define new sets $\Pi_{kl}$ so that they mutually disjoint and have the same cardinality as $B_{kl}$. In this way, the formula (A5) will be proved by establishing a set identity and counting their cardinality. To this end, we define $\Pi_{kl}:=\left\{\left(p,k,l\right):p\in B_{kl}\right\}$, for each $k,l\in Z_{N}$ with k≠l, and $\Pi_{p}:=\left\{\left(p,k,l\right):k,l\in B_{p}\;\mathrm{with}\;k\neq l\right\}$, for each $p\in Z_{N}$. From the definition of $\Pi_{kl}$, we have that $\#\Pi_{kl}=B_{kl}$ and $\Pi_{kl}⋂\Pi_{k^{\prime }l^{\prime }}=⌀$ if $\left(k,l\right)\neq \left(k^{\prime },l^{\prime }\right)$. Thus,

(A6) $$\sum_{k\in Z_{N}}\sum_{l\in Z_{N}\backslash \left\{k\right\}}B_{kl}=\#\left(\underset{k\in Z_{N}}{⋃}\underset{l\in Z_{N}\backslash \left\{k\right\}}{⋃}\Pi_{kl}\right).$$

Likewise, the definition of $\Pi_{p}$ ensures that $\#\Pi_{p}=B_{p}\left(B_{p}-1\right)$ and $\Pi_{p}⋂\Pi_{p^{\prime }}=⌀$ if $p\neq p^{\prime }$. Thus, by noting that $2B=\sum_{p\in Z_{N}}B_{p}$,

(A7) $$\sum_{p\in Z_{N}}B_{p}^{2}-2B=\sum_{p\in Z_{N}}B_{p}\left(B_{p}-1\right)=\#\left(\underset{p\in Z_{N}}{⋃}\Pi_{p}\right).$$

Combining Equations (A6) and (A7), we see that it suffices to prove

(A8) $$\underset{k\in Z_{N}}{⋃}\underset{l\in Z_{N}\backslash \left\{k\right\}}{⋃}\Pi_{kl}=\underset{p\in Z_{N}}{⋃}\Pi_{p}.$$

For all $k,l\in Z_{N}$ with k≠l, and $\left(p,k,l\right)\in \Pi_{kl}$, the definitions of $\Pi_{kl}$ and $B_{kl}$ ensure

(A9) $$p\neq k,\;p\neq l,\;\rho \left(y_{p},y_{k}\right)\leq r\;\;\mathrm{and}\;\rho \left(y_{p},y_{l}\right)\leq r.$$

In other words, there are $k\in B_{p}$, $l\in B_{p}$ and k≠l. Thus, for all $k,l\in Z_{N}$ with k≠l, and $\left(p,k,l\right)\in \Pi_{kl}$, there has $\left(p,k,l\right)\in \Pi_{p}$. Thus, we obtain

(A10) $$\underset{k\in Z_{N}}{⋃}\underset{l\in Z_{N}\backslash \left\{k\right\}}{⋃}\Pi_{kl}\subset \underset{p\in Z_{N}}{⋃}\Pi_{p}.$$

On the other hand, for all $p\in Z_{N}$ and $\left(p,k,l\right)\in \Pi_{p}$, we know (A9) holds and k≠l from the definitions of $\Pi_{p}$ and $B_{p}$. This means that $\left(p,k,l\right)\in \Pi_{kl}$ and k≠l. Hence, we obtain that

(A11) $$\underset{p\in Z_{N}}{⋃}\Pi_{p}\subset \underset{k\in Z_{N}}{⋃}\underset{l\in Z_{N}\backslash \left\{k\right\}}{⋃}\Pi_{kl}.$$

From (A10) and (A11) we obtain (A8), which leads to the desired result (A5). □

For $i,j\in Z_{N_{0}}$ with i≠j, we define random variable $Z_{ij}$ on the probability space {Ω,F,P} by

(A12) $$Z_{ij}\left(s\right):=\left\{\begin{array}{c} \;1,\;\mathrm{if}\;\rho (y_{s_{i}},y_{s_{j}})\leq r,\\ \;0,\;\mathrm{if}\;\rho (y_{s_{i}},y_{s_{j}})>r, \end{array}\right.\;\;s\in \Omega .$$

From the definition of ${\tilde{B}}_{j}$, we can see that ${\tilde{B}}_{j}=\sum_{i\in Z_{N_{0}}\backslash \left\{j\right\}}Z_{ij}$. Thus, in order to compute the covariance $E[{\tilde{B}}_{j_{1}}{\tilde{B}}_{j_{2}}]$, we next show the values of $P(Z_{i_{1}j_{1}}=1,Z_{i_{2}j_{2}}=1)$ for $j_{1},j_{2}\in Z_{N_{0}}$ and $i_{1},i_{2}\in Z_{N_{0}}\backslash \left\{j_{1},j_{2}\right\}$ with $i_{1}\neq i_{2}$.

**Lemma** **A3.**
*It holds that for $j_{1},j_{2}\in Z_{N_{0}}$ with $j_{1}\neq j_{2}$, and $i_{1},i_{2}\in Z_{N_{0}}\backslash \left\{j_{1},j_{2}\right\}$ with $i_{1}\neq i_{2}$,*

(A13)
$$P\left(Z_{i_{1}j_{1}}=1,Z_{i_{2}j_{2}}=1\right)=\frac{4\left(B^{2}+B-\sum_{l\in Z_{N}}B_{l}^{2}\right)}{N(N-1)(N-2)(N-3)}.$$

*Moreover, for all $j\in Z_{N_{0}}$ and $i,i^{\prime }\in Z_{N_{0}}\backslash \left\{j\right\}$ with $i\neq i^{\prime }$, it holds that*

(A14)
$$P\left(Z_{ij}=1,Z_{i^{\prime }j}=1\right)=\frac{\sum_{l\in Z_{N}}B_{l}^{2}-2B}{N(N-1)(N-2)}.$$

**Proof.** We first prove (A13). Let

$$L_{N_{0}}:=\left\{\left(i_{1},j_{1},i_{2},j_{2}\right):j_{1},j_{2}\in Z_{N_{0}}\;\mathrm{with}\;j_{1}\neq j_{2},\;\mathrm{and}\;i_{1},i_{2}\in Z_{N_{0}}\backslash \left\{j_{1},j_{2}\right\}\;\mathrm{with}\;i_{1}\neq i_{2}\right\}$$

and for all $\left(i_{1},j_{1},i_{2},j_{2}\right)\in L_{N_{0}}$, we define

$$\Omega_{i_{1}j_{1},i_{2}j_{2}}:=\left\{s\in \Omega :Z_{i_{1}j_{1}}\left(s\right)=1,\;\mathrm{and}\;Z_{i_{2}j_{2}}\left(s\right)=1\right\}.$$

We prove (A13) by counting the cardinality of $\Omega_{i_{1}j_{1},i_{2}j_{2}}$. To this end, we identify $\Omega_{i_{1}j_{1},i_{2}j_{2}}$ as the union of disjoint subsets of $\Omega_{i_{1}j_{1},i_{2}j_{2}}$. From the definition of $Z_{i_{1}j_{1}}$ and $Z_{i_{2}j_{2}}$, we know for all $\left(i_{1},j_{1},i_{2},j_{2}\right)\in L_{N_{0}}$ and $s\in \Omega_{i_{1}j_{1},i_{2}j_{2}}$ that $s_{i_{1}}\in B_{s_{j_{1}}}$ and $s_{i_{2}}\in B_{s_{j_{2}}}$. At the same time, note that for $\left(i_{1},j_{1},i_{2},j_{2}\right)\in L_{N_{0}}$ and $s\in \Omega_{i_{1}j_{1},i_{2}j_{2}}$, the numbers in set $\left\{s_{j_{1}},s_{j_{2}},s_{i_{1}},s_{i_{2}}\right\}$ are distinct. Thus, for all $\left(i_{1},j_{1},i_{2},j_{2}\right)\in L_{N_{0}}$ and $s\in \Omega_{i_{1}j_{1},i_{2}j_{2}}$, it holds that $s_{j_{1}}\neq s_{j_{2}}$, $s_{i_{1}}\in B_{s_{j_{1}}}\backslash \left\{s_{j_{2}}\right\}$, and $s_{i_{2}}\in B_{s_{j_{2}}}\backslash \left\{s_{i_{1}},s_{j_{1}}\right\}$. Namely,

$$\Omega_{i_{1}j_{1},i_{2}j_{2}}\subset \left\{s\in \Omega :s_{j_{1}}\neq s_{j_{2}},s_{i_{1}}\in B_{s_{j_{1}}}\backslash \left\{s_{j_{2}}\right\},\;\mathrm{and}\;s_{i_{2}}\in B_{s_{j_{2}}}\backslash \left\{s_{i_{1}},s_{j_{1}}\right\}\right\}.$$

On the other hand, it is easy to check that

$$\Omega_{i_{1}j_{1},i_{2}j_{2}}⊃\left\{s\in \Omega :s_{j_{1}}\neq s_{j_{2}},s_{i_{1}}\in B_{s_{j_{1}}}\backslash \left\{s_{j_{2}}\right\},\;\mathrm{and}\;s_{i_{2}}\in B_{s_{j_{2}}}\backslash \left\{s_{i_{1}},s_{j_{1}}\right\}\right\}.$$

Thus, for all $\left(i_{1},j_{1},i_{2},j_{2}\right)\in L_{N_{0}}$, $\Omega_{i_{1}j_{1},i_{2}j_{2}}$ can be rewritten as

$$\Omega_{i_{1}j_{1},i_{2}j_{2}}=\left\{s\in \Omega :s_{j_{1}}\neq s_{j_{2}},s_{i_{1}}\in B_{s_{j_{1}}}\backslash \left\{s_{j_{2}}\right\},\;\mathrm{and}\;s_{i_{2}}\in B_{s_{j_{2}}}\backslash \left\{s_{i_{1}},s_{j_{1}}\right\}\right\}.$$

For k≠l, we define $\Omega_{i_{1}j_{1},i_{2}j_{2}}^{k,l}:=\left\{s\in \Omega_{i_{1}j_{1},i_{2}j_{2}}:s_{j_{1}}=k,s_{j_{2}}=l\right\}.$ Then, we can rewrite $\Omega_{i_{1}j_{1},i_{2}j_{2}}$ as

(A15) $$\begin{array}{ccc} \Omega_{i_{1}j_{1},i_{2}j_{2}} & = & \underset{k\in Z_{N}}{⋃}\underset{l\in Z_{N}\backslash \left\{k\right\}}{⋃}\Omega_{i_{1}j_{1},i_{2}j_{2}}^{k,l}. \end{array}$$

Since $\Omega_{i_{1}j_{1},i_{2}j_{2}}^{k,l}\cap \Omega_{i_{1}j_{1},i_{2}j_{2}}^{k^{\prime },l^{\prime }}=⌀$ if $\left(k,l\right)\neq \left(k^{\prime },l^{\prime }\right)$, from (A15) we can see that

(A16) $$\begin{array}{c} \#\Omega_{i_{1}j_{1},i_{2}j_{2}}=\sum_{k\in Z_{N}}\sum_{l\in Z_{N}\backslash \left\{k\right\}}\#\Omega_{i_{1}j_{1},i_{2}j_{2}}^{k,l}. \end{array}$$

Note that for all $k\in Z_{N}$ and $l\in Z_{N}\backslash \left\{k\right\}$,

$$\begin{array}{ccc} \Omega_{i_{1}j_{1},i_{2}j_{2}}^{k,l} & = & \left\{s\in \Omega :s_{j_{1}}=k,s_{j_{2}}=l,s_{i_{1}}\in B_{k}\backslash \left(B_{kl}\cup \left\{l\right\}\right)\;\mathrm{and}\;s_{i_{2}}\in B_{l}\backslash \left\{k\right\}\right\}\\ & & \cup \{s\in \Omega :s_{j_{1}}=k,s_{j_{2}}=l,s_{i_{1}}\in B_{kl}\;\mathrm{and}\;s_{i_{2}}\in B_{l}\backslash \left\{s_{i_{1}},k\right\}\}, \end{array}$$

and the two sets on the right-hand side of the above equation are disjoint. Thus, it holds that for all $k\in Z_{N}$ and $l\in Z_{N}\backslash \left\{k\right\}$,

(A17) $$\begin{array}{c} \#\Omega_{i_{1}j_{1},i_{2}j_{2}}^{k,l}=\frac{(N-4)!}{(N-N_{0})!}\left(\left(B_{k}-B_{kl}-Z_{kl}\right)\left(B_{l}-Z_{kl}\right)+B_{kl}\left(B_{l}-Z_{kl}-1\right)\right). \end{array}$$

Substituting (A17) into (A16) leads to

$$\#\Omega_{i_{1}j_{1},i_{2}j_{2}}=\frac{(N-4)!}{(N-N_{0})!}\sum_{k\in Z_{N}}\sum_{l\in Z_{N}\backslash \left\{k\right\}}\left(\left(B_{k}-B_{kl}-Z_{kl}\right)\left(B_{l}-Z_{kl}\right)+B_{kl}\left(B_{l}-Z_{kl}-1\right)\right).$$

By direct computation with noting $Z_{kl}^{2}=Z_{kl}$, we obtain from the equation above that

(A18) $$\#\Omega_{i_{1}j_{1},i_{2}j_{2}}=\frac{(N-4)!}{(N-N_{0})!}\sum_{k\in Z_{N}}\sum_{l\in Z_{N}\backslash \left\{k\right\}}\left(B_{k}B_{l}-B_{k}Z_{kl}-B_{l}Z_{kl}-B_{kl}+Z_{kl}\right).$$

Note that $\sum_{k\in Z_{N}\backslash \left\{l\right\}}Z_{kl}=B_{l}$ and $\sum_{l\in Z_{N}\backslash \left\{k\right\}}B_{l}=2B-B_{k}$. We then have that

$$\sum_{k\in Z_{N}}\sum_{l\in Z_{N}\backslash \left\{k\right\}}B_{l}Z_{kl}=\sum_{l\in Z_{N}}\sum_{k\in Z_{N}\backslash \left\{l\right\}}B_{l}Z_{kl}=\sum_{l\in Z_{N}}B_{l}\left(\sum_{k\in Z_{N}\backslash \left\{l\right\}}Z_{kl}\right)=\sum_{l\in Z_{N}}B_{l}^{2},$$

$$\sum_{k\in Z_{N}}\sum_{l\in Z_{N}\backslash \left\{k\right\}}B_{k}B_{l}=\sum_{k\in Z_{N}}B_{k}\left(2B-B_{k}\right)=4B^{2}-\sum_{l\in Z_{N}}B_{l}^{2},$$

and

$$\sum_{k\in Z_{N}}\sum_{l\in Z_{N}\backslash \left\{k\right\}}Z_{kl}=2B.$$

Substituting (A5) and the above equations into (A18), we obtain that

(A19) $$\begin{array}{c} \#\Omega_{i_{1}j_{1},i_{2}j_{2}}=\frac{4(N-4)!}{(N-N_{0})!}\left(B^{2}+B-\sum_{l\in Z_{N}}B_{l}^{2}\right). \end{array}$$

By noting that $\#\Omega =\frac{N!}{(N-N_{0})!}$ and $P\left(Z_{i_{1}j_{1}}=1,Z_{i_{2}j_{2}}=1\right)=\frac{\#\Omega_{i_{1}j_{1},i_{2}j_{2}}}{\#\Omega },$ we obtain (A13) from (A19). We now turn to prove (A14). Let $j\in Z_{N_{0}}$ and $i,i^{\prime }\in Z_{N_{0}}\backslash \left\{j\right\}$ with $i\neq i^{\prime }$. Note that

$$\begin{array}{ccc} \#\{s\in \Omega :Z_{ij}\left(s\right)=Z_{i^{\prime }j}\left(s\right)=1\} & = & \sum_{l\in Z_{N}}\#\left\{s\in \Omega :s_{j}=l,s_{i}\in B_{l}\;\mathrm{and}\;s_{i^{\prime }}\in B_{l}\backslash \left\{s_{i}\right\}\right\}\\ & = & \frac{(N-3)!}{(N-N_{0})!}\sum_{l\in Z_{N}}B_{l}\left(B_{l}-1\right). \end{array}$$

Thus, it holds that

(A20) $$P\left(Z_{ij}=1,Z_{i^{\prime }j}=1\right)=\frac{\sum_{l\in Z_{N}}B_{l}\left(B_{l}-1\right)}{N(N-1)(N-2)}.$$

Since $\sum_{l\in Z_{N}}B_{l}=2B$, from (A20) we obtain (A14). □

With the help of Lemma A3, we can calculate $E\left[{\tilde{B}}_{j_{1}}{\tilde{B}}_{j_{2}}\right]$ in the following lemma.

**Lemma** **A4.**
*If $N_{0}\in Z_{N}$ with $N_{0}>3$, then for all $j_{1},j_{2}\in Z_{N_{0}}$ with $j_{1}\neq j_{2}$,*

(A21)
$$\begin{array}{cc} E\left[{\tilde{B}}_{j_{1}}{\tilde{B}}_{j_{2}}\right]\; & =\frac{4\left(N_{0}-2\right)\left(N_{0}-3\right)}{N(N-1)(N-2)(N-3)}\left(B^{2}+B-\sum_{l\in Z_{N}}B_{l}^{2}\right)\\ \; & +\frac{3\left(N_{0}-2\right)\left(\sum_{l\in Z_{N}}B_{l}^{2}-2B\right)}{N(N-1)(N-2)}+\frac{2B}{N(N-1)}, \end{array}$$

*and for all $j\in Z_{N_{0}}$,*

(A22)
$$E\left[{\tilde{B}}_{j}^{2}\right]=\frac{2(N_{0}-1)B}{N(N-1)}+\frac{\left(N_{0}-1\right)\left(N_{0}-2\right)}{N(N-1)(N-2)}\left(\sum_{l\in Z_{N}}B_{l}^{2}-2B\right).$$

**Proof.** We first prove (A21). Let $j_{1},j_{2}\in Z_{N_{0}}$ with $j_{1}\neq j_{2}$. From the decomposition ${\tilde{B}}_{j}=\sum_{i\in Z_{N_{0}}\backslash \left\{j\right\}}Z_{ij}$, we obtain for all $j_{1},j_{2}\in Z_{N_{0}}$ with $j_{1}\neq j_{2}$ that

$$E\left[{\tilde{B}}_{j_{1}}{\tilde{B}}_{j_{2}}\right]=\sum_{i_{1}\in Z_{N_{0}}\backslash \left\{j_{1}\right\}}\sum_{i_{2}\in Z_{N_{0}}\backslash \left\{j_{2}\right\}}E\left[Z_{i_{1}j_{1}}Z_{i_{2}j_{2}}\right].$$

We further rewrite the right-hand side of the above equation to obtain

(A23) $$\begin{array}{ccc} E\left[{\tilde{B}}_{j_{1}}{\tilde{B}}_{j_{2}}\right] & = & \sum_{i_{1}\in Z_{N_{0}}\backslash \left\{j_{1},j_{2}\right\}}\sum_{i_{2}\in Z_{N_{0}}\backslash \left\{j_{1},j_{2},i_{1}\right\}}E\left[Z_{i_{1}j_{1}}Z_{i_{2}j_{2}}\right]\\ & & +\sum_{i_{1}\in Z_{N_{0}}\backslash \left\{j_{1},j_{2}\right\}}E\left[Z_{i_{1}j_{1}}Z_{i_{1}j_{2}}\right]+\sum_{i_{1}\in Z_{N_{0}}\backslash \left\{j_{1},j_{2}\right\}}E\left[Z_{i_{1}j_{1}}Z_{j_{1}j_{2}}\right]\\ & & +\sum_{i_{2}\in Z_{N_{0}}\backslash \left\{j_{1},j_{2}\right\}}E\left[Z_{j_{2}j_{1}}Z_{i_{2}j_{2}}\right]+E\left[Z_{j_{2}j_{1}}Z_{j_{1}j_{2}}\right]. \end{array}$$

We next compute the terms on the right hand side of (A23) one by one. Since for all $j,j^{\prime }\in Z_{N_{0}}$, $i\in Z_{N_{0}}\backslash \left\{j\right\}$ and $i^{\prime }\in Z_{N_{0}}\backslash \left\{j^{\prime }\right\}$,

$$E\left[Z_{ij}Z_{i^{\prime }j^{\prime }}\right]=P\left(Z_{ij}=1,Z_{i^{\prime }j^{\prime }}=1\right),$$

from Equation (A13) of Lemma A3, we know the first term in the right-hand side of (A23) satisfies

(A24) $$\sum_{i_{1}\in Z_{N_{0}}\backslash \left\{j_{1},j_{2}\right\}}\sum_{i_{2}\in Z_{N_{0}}\backslash \left\{j_{1},j_{2},i_{1}\right\}}E\left[Z_{i_{1}j_{1}}Z_{i_{2}j_{2}}\right]=\frac{4\left(B^{2}+B-\sum_{l\in Z_{N}}B_{l}^{2}\right)}{N(N-1)(N-2)(N-3)}\left(N_{0}-2\right)\left(N_{0}-3\right).$$

Likewise, by noting that $Z_{ij}=Z_{ji}$, from Equation (A14) of Lemma A3, we obtain the second, third, and fourth terms on the right-hand side of (A23),

(A25) $$\begin{array}{ccc} \sum_{i_{1}\in Z_{N_{0}}\backslash \left\{j_{1},j_{2}\right\}}E\left[Z_{i_{1}j_{1}}Z_{i_{1}j_{2}}\right] & = & \sum_{i_{1}\in Z_{N_{0}}\backslash \left\{j_{1},j_{2}\right\}}E\left[Z_{i_{1}j_{1}}Z_{j_{1}j_{2}}\right]=\sum_{i_{2}\in Z_{N_{0}}\backslash \left\{j_{1},j_{2}\right\}}E\left[Z_{j_{2}j_{1}}Z_{i_{2}j_{2}}\right]\\ & = & \left(N_{0}-2\right)\frac{\sum_{l\in Z_{N}}B_{l}^{2}-2B}{N(N-1)(N-2)}. \end{array}$$

Note that for all $i,j\in Z_{N_{0}}$ with i≠j, it holds that $Z_{ij}=Z_{ji}$ and $Z_{ij}^{2}=Z_{ij}$. Thus, the last term on the right-hand side of (A23) satisfies

(A26) $$E\left[Z_{j_{2}j_{1}}Z_{j_{1}j_{2}}\right]=\frac{2B}{N(N-1)}.$$

Substituting (A24), (A25), and (A26) into (A23) leads to (A21). It remains to prove (A22). Since for all $j\in Z_{N_{0}}$, ${\tilde{B}}_{j}=\sum_{i\in Z_{N_{0}}\backslash \left\{j\right\}}Z_{ij}=\sum_{i\in Z_{N_{0}}\backslash \left\{j\right\}}Z_{ij}^{2}$, there has

(A27) $$\begin{array}{ccc} E\left[{\tilde{B}}_{j}^{2}\right] & = & E\left[\sum_{i\in Z_{N_{0}}\backslash \left\{j\right\}}Z_{ij}^{2}+\sum_{i_{1}\in Z_{N_{0}}\backslash \left\{j\right\}}\sum_{i_{2}\in Z_{N_{0}}\backslash \left\{j,i_{1}\right\}}Z_{i_{1}j}Z_{i_{2}j}\right],\\ & = & E\left[{\tilde{B}}_{j}+\sum_{i_{1}\in Z_{N_{0}}\backslash \left\{j\right\}}\sum_{i_{2}\in Z_{N_{0}}\backslash \left\{j,i_{1}\right\}}Z_{i_{1}j}Z_{i_{2}j}\right]\\ & = & E\left[{\tilde{B}}_{j}\right]+\sum_{i_{1}\in Z_{N_{0}}\backslash \left\{j\right\}}\sum_{i_{2}\in Z_{N_{0}}\backslash \left\{j,i_{1}\right\}}E\left[Z_{i_{1}j}Z_{i_{2}j}\right]. \end{array}$$

Note that for all $j\in Z_{N_{0}}$, $i_{1}\in Z_{N_{0}}\backslash \left\{j\right\}$ and $i_{2}\in Z_{N_{0}}\backslash \left\{j,i_{1}\right\}$,

$$E\left[Z_{i_{1}j}Z_{i_{2}j}\right]=P\left(Z_{i_{1}j}=1,Z_{i_{2}j}=1\right).$$

Thus, substituting (A2) and (A14) into (A27), we obtain (A22). □

Now, we are ready to discuss the variance of $\frac{\tilde{B}}{N_{0}\left(N_{0}-1\right)}$.

**The proof for Theorem 4 is shown as follows.**

**Proof.** To prove this theorem, we compute $E[{\tilde{B}}^{2}]$. Noting that $\tilde{B}=\frac{1}{2}\sum_{j=1}^{N_{0}}{\tilde{B}}_{j}$, we have

(A28) $$E\left[{\tilde{B}}^{2}\right]=\frac{1}{4}\left(\sum_{j\in Z_{N_{0}}}E\left[{\tilde{B}}_{j}^{2}\right]+\sum_{j_{1}\in Z_{N_{0}}}\sum_{j_{2}\in Z_{N_{0}}\backslash \left\{j_{1}\right\}}E\left[{\tilde{B}}_{j_{1}}{\tilde{B}}_{j_{2}}\right]\right).$$

Substituting (A21) and (A22) into (A28) leads to

(A29) $$\begin{array}{c} E\left[{\tilde{B}}^{2}\right]=\frac{N_{0}\left(N_{0}-1\right)}{N(N-1)}B+\frac{N_{0}\left(N_{0}-1\right)\left(N_{0}-2\right)}{N(N-1)(N-2)}\left(\sum_{p=1}^{N}B_{p}^{2}-2B\right)\\ +\frac{N_{0}\left(N_{0}-1\right)\left(N_{0}-2\right)\left(N_{0}-3\right)}{N(N-1)(N-2)(N-3)}\left(B^{2}-\sum_{p=1}^{N}B_{p}^{2}+B\right). \end{array}$$

Since

$$\mathrm{Var}\left[\frac{\tilde{B}}{N_{0}\left(N_{0}-1\right)}\right]=E\left[{\left(\frac{\tilde{B}}{N_{0}\left(N_{0}-1\right)}\right)}^{2}\right]-{\left(E\left[\frac{\tilde{B}}{N_{0}\left(N_{0}-1\right)}\right]\right)}^{2},$$

by conducting some computation, from (A29) and the definition of $C_{N_{0}}$ (5), we obtain (4). We next estimate $C_{N_{0}}$. It can be checked that

(A30) $$\begin{array}{ccc} C_{N_{0}} & = & \frac{\left(N_{0}-2\right)\left(\sum_{l\in Z_{N}}B_{l}^{2}\right)}{\left(N_{0}-1\right)\left(N-1\right)N\left(N-2\right)}\left(1-\frac{N_{0}-3}{N-3}\right)\\ & & +\frac{B}{\left(N_{0}-1\right)N\left(N-1\right)}\left(1-2\frac{N_{0}-2}{N-2}+\frac{\left(N_{0}-2\right)\left(N_{0}-3\right)}{\left(N-2)(N-3\right)}\right)\\ & & +\frac{B^{2}}{N\left(N-1\right)\left(N_{0}-1\right)}\left(\frac{\left(N_{0}-2\right)\left(N_{0}-3\right)}{\left(N-2)(N-3\right)}-\frac{N_{0}\left(N_{0}-1\right)}{N(N-1)}\right). \end{array}$$

By noting $\frac{\sum_{l\in Z_{N}}B_{l}^{2}}{{\left(N-1\right)}^{2}N}\leq 1$, $\frac{\left(N_{0}-2\right)\left(N-1\right)}{\left(N_{0}-1\right)\left(N-2\right)}\leq 1$ and $0\leq 1-\frac{N_{0}-3}{N-3}\leq 1$, we know the first term in (A30) is not greater than 1. Since $\frac{B}{N(N-1)}<\frac{1}{2}$ and $1-2\frac{N_{0}-2}{N-2}+\frac{\left(N_{0}-2\right)\left(N_{0}-3\right)}{\left(N-2)(N-3\right)}\leq {\left(1-\frac{N_{0}-2}{N-2}\right)}^{2}<1$, we have that the second term in (A30) is not greater than $\frac{1}{N_{0}-1}$. Note that $\frac{\left(N_{0}-2\right)\left(N_{0}-3\right)}{\left(N-2)(N-3\right)}-\frac{N_{0}\left(N_{0}-1\right)}{N(N-1)}\leq 0$. Thus, we know the third term in (A30) is not positive. Therefore, we conclude that $C_{N_{0}}\leq 1+\frac{1}{2(N_{0}-1)}$. □

To analyze this almost sure convergence rate of $\left\{-\log\frac{{\bar{B}}_{k}}{{\bar{A}}_{k}}:k\in N\right\}$, we require Theorem 2 of [18], which is recalled as follows.

**Theorem** **A2.**
*Let $\left\{V_{i}:i\in N\right\}\cup \left\{V\right\}$ be a sequence of independent and identically distributed random variables in probability space {Ω,F,P} with expectation μ, $\sigma :=\mathrm{Var}[V_{i}]$ and $\tau :=E\left[|V_{i}-\mu |\right]$. If σ<+∞ and τ<+∞, then for all 0<ϵ≤1 and β>1, there are constants $D_{\beta }$ and ${\tilde{D}}_{\beta }$ (depending only on β) such that for all $i>n_{\epsilon ,\beta }$,*

$$P\left(\underset{k\geq i}{\sup}\left|\frac{1}{k}\sum_{l=1}^{k}V_{l}-\mu \right|>\tau \epsilon \right)\leq \frac{72\sigma }{\tau^{2}\epsilon^{2}i}\left(D_{\beta }+{\tilde{D}}_{\beta }{\left(\log i\right)}^{\beta -1}\right),$$

*where $n_{\epsilon ,\beta }$ is defined by (6).*

Combining Theorems 3, 4, and A2 leads to the almost sure convergence of $\frac{{\bar{B}}_{N_{1}}}{N_{0}\left(N_{0}-1\right)}$ and $\frac{{\bar{A}}_{N_{1}}}{N_{0}\left(N_{0}-1\right)}$ in the next lemma.

**Lemma** **A5.**
*Let β>1 and $N_{0}\in Z_{N}$ with $N_{0}>3$. Then, there are constants $D_{\beta }$ and ${\tilde{D}}_{\beta }$ (depending only on β) such that for all 1≥ϵ>0 and $N_{1}>n_{\epsilon ,\beta }$,*

(A31)
$$P\left(\underset{k>N_{1}}{\sup}\left|\frac{{\bar{A}}_{k}}{N_{0}\left(N_{0}-1\right)}-\frac{A}{N(N-1)}\right|>\tau_{A}\epsilon \right)\leq \frac{72C_{N_{0}}}{\tau_{A}^{2}\epsilon^{2}N_{0}N_{1}}\left(D_{\beta }+{\tilde{D}}_{\beta }{\left(\log N_{1}\right)}^{\beta -1}\right),$$

*and*

(A32)
$$P\left(\underset{k>N_{1}}{\sup}\left|\frac{{\bar{B}}_{k}}{N_{0}\left(N_{0}-1\right)}-\frac{B}{N(N-1)}\right|>\tau_{B}\epsilon \right)\leq \frac{72C_{N_{0}}}{\tau_{B}^{2}\epsilon^{2}N_{0}N_{1}}\left(D_{\beta }+{\tilde{D}}_{\beta }{\left(\log N_{1}\right)}^{\beta -1}\right),$$

*where $n_{\epsilon ,\beta }$ is defined by (6).*

**Proof.** From Theorems 3 and 4, we know that $\mathrm{Var}\left[\frac{\tilde{A}}{N_{0}\left(N_{0}-1\right)}\right]=\mathrm{Var}\left[\frac{\tilde{B}}{N_{0}\left(N_{0}-1\right)}\right]=\frac{C_{N_{0}}}{N_{0}}<+\infty$, $E\left[\frac{\tilde{A}}{N_{0}\left(N_{0}-1\right)}\right]=\frac{A}{N(N-1)}$, and $E\left[\frac{\tilde{B}}{N_{0}\left(N_{0}-1\right)}\right]=\frac{B}{N(N-1)}$. Meanwhile, since $0\leq \frac{\tilde{A}}{N_{0}\left(N_{0}-1\right)}\leq 1$, $0\leq \frac{\tilde{B}}{N_{0}\left(N_{0}-1\right)}\leq 1$, $0\leq \frac{A}{N(N-1)}\leq 1$ and $0\leq \frac{B}{N(N-1)}\leq 1$, we know that $\tau_{A}\leq 1$ and $\tau_{B}\leq 1$. Thus, by Theorem A2, we obtain (A32) and (A31). □

We next consider the almost sure convergence rate of $\left\{-\log\frac{{\bar{B}}_{k}}{{\bar{A}}_{k}}:k\in N\right\}$. To this end, we introduce the following lemma.

**Lemma** **A6.**
*Let $N_{0}\in Z_{N}$ with $N_{0}>3$. If A>0 and B>0, then for all $N_{1}\in N$ and 1>ϵ>0,*

(A33)
$$\begin{array}{ccc} & & P\left(\underset{k>N_{1}}{\sup}\left|\log\left(\frac{{\bar{A}}_{k}}{N_{0}\left(N_{0}-1\right)}\right)-\log\left(\frac{A}{N(N-1)}\right)\right|>\epsilon \right)\\ & \leq & P\left(\underset{k>N_{1}}{\sup}\left|\frac{{\bar{A}}_{k}}{N_{0}\left(N_{0}-1\right)}-\frac{A}{N(N-1)}\right|>\frac{A\epsilon }{N(N-1)e}\right), \end{array}$$

*and*

(A34)
$$\begin{array}{ccc} & & P\left(\underset{k>N_{1}}{\sup}\left|\log\left(\frac{{\bar{B}}_{k}}{N_{0}\left(N_{0}-1\right)}\right)-\log\left(\frac{B}{N(N-1)}\right)\right|>\epsilon \right)\\ & \leq & P\left(\underset{k>N_{1}}{\sup}\left|\frac{{\bar{B}}_{k}}{N_{0}\left(N_{0}-1\right)}-\frac{B}{N(N-1)}\right|>\frac{B\epsilon }{N(N-1)e}\right). \end{array}$$

**Proof.** Note that for all 0<a,b<1 and 0<η<1, when

(A35) $$\left|\log a-\log b\right|>\eta ,$$

it holds that $a>be^{\eta },\;\mathrm{or}\;a<be^{-\eta }.$ Hence, when (A35) holds, there is

(A36) $$a-b>b\left(e^{\eta }-1\right),\;\mathrm{or}\;a-b<b\left(e^{-\eta }-1\right).$$

By noting that $e^{\eta }-1>1-e^{-\eta }$ and $1-e^{-\eta }>e^{-1}\eta$, from (A36), we know that when (A35) holds, there has $a-b>be^{-1}\eta ,\;\mathrm{or}\;a-b<-be^{-1}\eta ,$ that is,

(A37) $$\left|a-b\right|>be^{-1}\eta .$$

Note that when a=0, for all 0<b<1 and 0<η<1, inequality (A37) always holds. Thus, we know that for all 0≤a<1 and 0<b,η<1, when (A35) holds, inequality (A37) holds. Then, replacing *a*, *b*, and η by $\frac{{\bar{B}}_{k}}{N_{0}\left(N_{0}-1\right)}$, $\frac{B}{N(N-1)}$ and ϵ, we know for all $N_{1}\in N$ and 0<ϵ<1, when

(A38) $$\underset{k>N_{1}}{\sup}\left|\log\left(\frac{{\bar{B}}_{k}}{N_{0}\left(N_{0}-1\right)}\right)-\log\left(\frac{B}{N(N-1)}\right)\right|>\epsilon ,$$

there has

(A39) $$\underset{k>N_{1}}{\sup}\left|\frac{{\bar{B}}_{k}}{N_{0}\left(N_{0}-1\right)}-\frac{B}{N(N-1)}\right|>\frac{B\epsilon }{N(N-1)e}.$$

Let $F_{1}$ be the set of the events satisfying (A38), and $F_{2}$ be the set of the events satisfying (A39). From (A38) and (A39), we know that $F_{1}\subset F_{2}$. Thus, we can obtain (A34) (see Theorem 1.5.4 in [29]). Similarly, we can obtain (A33). □

Combining Lemmas A5 and A6, we obtain the almost sure convergence rate of

$$\left\{-\log\frac{{\bar{B}}_{k}}{{\bar{A}}_{k}}:k\in N\right\}$$

in Theorem 5.

**The proof of Theorem 5 is provided as follows.**

**Proof.** Note that for all $N_{1}\in N$,

$$\begin{array}{ccc} \underset{k>N_{1}}{\sup}\left|\log\frac{{\bar{B}}_{k}}{{\bar{A}}_{k}}-\log\frac{B}{A}\right| & \leq & \underset{k>N_{1}}{\sup}\left|\log\left(\frac{{\bar{B}}_{k}}{N_{0}\left(N_{0}-1\right)}\right)-\log\left(\frac{B}{N(N-1)}\right)\right|\\ & & +\underset{k>N_{1}}{\sup}\left|\log\left(\frac{{\bar{A}}_{k}}{N_{0}\left(N_{0}-1\right)}\right)-\log\left(\frac{A}{N(N-1)}\right)\right|. \end{array}$$

Thus, we know that for all $N_{1}\in N$ and 1>ϵ>0, if

(A40) $$\underset{k>N_{1}}{\sup}\left|\log\frac{{\bar{B}}_{k}}{{\bar{A}}_{k}}-\log\frac{B}{A}\right|>\max\left\{\tau_{A},\tau_{B}\right\}\epsilon ,$$

then

(A41) $$\underset{k>N_{1}}{\sup}\left|\log\left(\frac{{\bar{B}}_{k}}{N_{0}\left(N_{0}-1\right)}\right)-\log\left(\frac{B}{N(N-1)}\right)\right|>\frac{\max\{\tau_{A},\tau_{B}\}\epsilon }{2}\geq \frac{\tau_{B}\epsilon }{2},$$

or

(A42) $$\underset{k>N_{1}}{\sup}\left|\log\left(\frac{{\bar{A}}_{k}}{N_{0}\left(N_{0}-1\right)}\right)-\log\left(\frac{A}{N(N-1)}\right)\right|>\frac{\max\{\tau_{A},\tau_{B}\}\epsilon }{2}\geq \frac{\tau_{A}\epsilon }{2}.$$

Let $F_{1}$ be the set of the events satisfying (A40), $F_{2}$ be the set of the events satisfying (A41), and $F_{3}$ be the set of events satisfying (A42). Then, from the above inequalities, we have $F_{1}\subset F_{2}\cup F_{3}$. Hence, we have $P\left(F_{1}\right)\leq P\left(F_{2}\right)+P\left(F_{3}\right)$ (see Theorems 1.5.4 and 1.5.7 in [29]), that is,

$$\begin{array}{ccc} & & P\left(\underset{k>N_{1}}{\sup}\left|\log\frac{{\bar{B}}_{k}}{{\bar{A}}_{k}}-\log\frac{B}{A}\right|>\max\left\{\tau_{A},\tau_{B}\right\}\epsilon \right)\\ & \leq & P\left(\underset{k>N_{1}}{\sup}\left|\log\left(\frac{{\bar{B}}_{k}}{N_{0}\left(N_{0}-1\right)}\right)-\log\left(\frac{B}{N(N-1)}\right)\right|>\frac{\tau_{B}\epsilon }{2}\right)\\ & & +P\left(\underset{k>N_{1}}{\sup}\left|\log\left(\frac{{\bar{A}}_{k}}{N_{0}\left(N_{0}-1\right)}\right)-\log\left(\frac{A}{N(N-1)}\right)\right|>\frac{\tau_{A}\epsilon }{2}\right). \end{array}$$

Substituting (A34) and (A33) into above inequality, from Lemma A5 and the definitions of $\gamma_{A}$ and $\gamma_{B}$, we obtain the desired result (7). □
